# CAR-HEMATOTOX score as a predictor of hematotoxicity, infection, and survival after CAR T-cell therapy in hematologic malignancies: a systematic review and meta-analysis

**DOI:** 10.3389/fonc.2026.1928438

**Published:** 2026-09-01

**Authors:** Juncheng Wei, Ziyu Chen, Mengfei Li, Miao Jiang

**Affiliations:** 1 Dongzhimen Hospital, Beijing University of Chinese Medicine, Beijing, China; 2 College of Life Sciences, Beijing University of Chinese Medicine, Beijing, China

**Keywords:** CAR T-cell therapy, CAR-HEMATOTOX, hematologic malignancies, immune effector cell-associated hematotoxicity, infection, meta-analysis, survival

## Abstract

**Background:**

Immune effector cell-associated hematotoxicity (ICAHT) is a common and clinically important toxicity after chimeric antigen receptor (CAR) T-cell therapy. The CAR-HEMATOTOX score, also reported as CAR-HT, is a baseline risk score proposed to identify patients at higher risk of hematotoxicity and related adverse outcomes.

**Methods:**

We conducted a systematic search of PubMed, the Web of Science, and the Cochrane Library on June 19, 2026. Included studies compared high- and low-risk groups defined by the CAR-HEMATOTOX score after CAR T-cell therapy for hematologic malignancies and reported extractable data on hematologic toxicity, infection, survival, or other predefined clinical outcomes. Random-effects inverse-variance meta-analysis was performed only when at least three independent, clinically compatible estimates were available.

**Results:**

Twenty-three publications were included in the evidence inventory. For ICAHT, high risk was associated with higher odds (pooled OR 5.58, 95% CI 1.98 to 15.73; I²=60.8%). For overall survival (OS), high risk was associated with worse OS (pooled HR 3.36, 95% CI 2.20 to 5.11; I²=0.0%). For progression-free survival (PFS), high risk was associated with worse PFS (pooled HR 2.74, 95% CI 1.81 to 4.15; I²=32.3%). For severe infection, high-risk status was associated with higher odds (pooled OR 5.18, 95% CI 2.78 to 9.67; I²=37.8%). Exploratory analyses suggested higher early red blood cell and platelet transfusion requirements in high-risk patients, whereas severe cytokine release syndrome (CRS) and immune effector cell-associated neurotoxicity syndrome (ICANS) were not significantly associated with high-risk status.

**Conclusion:**

A high CAR-HEMATOTOX score was associated with greater risk of ICAHT, higher odds of severe infection, and worse survival outcomes after CAR T-cell therapy for hematologic malignancies. These findings suggest that the CAR-HEMATOTOX score may be a clinically relevant risk-stratification marker, but the evidence remains observational and exploratory; standardized outcome definitions, prospective validation, and clearer handling of overlapping cohorts are needed before the score can be used as a stand-alone decision tool.

**Systematic Review Registration:**

https://www.crd.york.ac.uk/prospero/view/CRD420261435303, identifier 420261435303.

## Introduction

CAR T-cell therapy has become an important treatment option for hematologic malignancies, but hematologic toxicity remains a frequent and clinically relevant complication. These events may include early or delayed cytopenias, prolonged blood-count recovery, transfusion requirements, and increased infection risk. Because hematotoxicity can extend beyond the acute cytokine-release period, ICAHT has provided a structured framework for describing these complications after immune effector cell therapy (1, 2).

The CAR-HEMATOTOX score was developed as a pre-lymphodepletion risk score based on markers of inflammation and hematopoietic reserve. It is commonly used to classify patients into high- and low-risk groups, most often with a cutoff of at least 2 points for high risk (3). Because the score reflects both inflammatory burden and marrow reserve, it may be associated not only with ICAHT but also with infection, transfusion needs, and survival outcomes (3, 5, 18). However, available studies differ in disease setting, CAR T-cell product, outcome definition, effect metric, and adjustment strategy (3, 5, 10).

Several related or comparator tools have been proposed, but they are not interchangeable with the original CAR-HEMATOTOX score (3, 7, 9). Previous work has also evaluated broader CAR T-cell adverse-event prediction, including model-performance outcomes (4). In contrast, the clinical association between high- and low-risk groups defined by the original CAR-HEMATOTOX score and interpretable outcomes remains incompletely summarized.

We therefore conducted a systematic review and outcome-specific meta-analysis to evaluate whether a high CAR-HEMATOTOX score is associated with ICAHT, infection, survival, and other clinically relevant outcomes after CAR T-cell therapy for hematologic malignancies. Quantitative synthesis was restricted to outcomes with independent and clinically compatible estimates, whereas less comparable outcomes were summarized narratively or evaluated in exploratory analyses where appropriate.

## Methods

### Design and registration

The protocol for this review has been prospectively registered with PROSPERO (Registration number: 420261435303), and the current manuscript was prepared following the PRISMA guidelines. The PRISMA checklist is provided in **Supplementary Note A1**.

### Data sources and search strategy

On June 19, 2026, we systematically searched PubMed, Web of Science, and the Cochrane Library. The search strategy incorporated terms related to CAR-HEMATOTOX, CAR T-cell therapy, hematologic malignancies, hematologic toxicity or cytopenia, infection, survival, mortality, and other clinically relevant outcomes. There were no restrictions on publication year. The language was limited to English. Full search strategies for each database are detailed in **Supplementary Note A2**.

### Eligibility criteria

Eligible studies met the following criteria: (1) included patients with hematologic malignancies treated with CAR T-cell therapy; (2) evaluated the original CAR-HEMATOTOX score and classified patients into high- and low-risk groups based on their baseline score; and (3) reported extractable data for at least one eligible outcome. Eligible outcomes included ICAHT or other hematotoxicity measures, infection, OS, PFS, non-relapse mortality or treatment-related mortality, hospitalization or supportive-care outcomes, CRS, ICANS, treatment response, and model-performance outcomes.

For quantitative synthesis, studies were required to report an independent and clinically compatible effect estimate, or sufficient event-count data to reconstruct an effect estimate. Studies evaluating only modified, simplified, or comparator risk tools were not pooled with studies of the original CAR-HEMATOTOX score unless results for the original score were reported separately. Reviews, editorials, protocols, conference abstracts without sufficient extractable data, studies not involving CAR T-cell therapy, and studies without relevant data linking the score to eligible outcomes were excluded. Studies that reported relevant outcome data but were not suitable for quantitative pooling were retained and summarized narratively where appropriate.

### Definition of the CAR-HEMATOTOX score

The CAR-HEMATOTOX score is calculated before lymphodepleting chemotherapy using five baseline laboratory variables: platelet count, absolute neutrophil count, hemoglobin, C-reactive protein, and ferritin (3). Points are assigned as follows: platelet count >175 × 10^9/L, 0 points; >75 to ≤175 × 10^9/L, 1 point; and ≤75 × 10^9/L, 2 points; ANC >1.2 × 10^9/L, 0 points, and ≤1.2 × 10^9/L, 1 point; hemoglobin >9.0 g/dL, 0 points, and ≤9.0 g/dL, 1 point; CRP <3.0 mg/dL, 0 points, and ≥3.0 mg/dL, 1 point; and ferritin <650 ng/mL, 0 points; ≥650 to <2,000 ng/mL, 1 point; and ≥2,000 ng/mL, 2 points. The individual points are summed to produce a total score ranging from 0 to 7. Patients with a score of 0–1 are classified as low risk, whereas those with a score of ≥2 are classified as high risk. In this review, risk-group classifications were extracted as reported in the included studies. The timing of laboratory assessment and any deviation from the original scoring definition were also recorded.

### Study selection

After database export, duplicate records were removed. Two reviewers independently screened titles and abstracts, followed by full-text assessment of potentially eligible studies. Records with insufficient information at the title or abstract stage were advanced to full-text review. Disagreements were resolved by discussion or, when necessary, by consultation with a third reviewer.

### Data extraction

Data were extracted independently by two reviewers using a predefined extraction form. Extracted data included study and treatment characteristics, CAR-HEMATOTOX score definitions, outcome definitions and time points, effect estimates, adjustment details, and event counts when available. We also recorded whether patients had undergone allogeneic hematopoietic stem cell transplantation (allo-HSCT) before CAR T-cell therapy or proceeded to allo-HSCT afterward, together with any reported analytical handling of these patients, including exclusion, censoring, or covariate adjustment. For binary outcomes, event numbers and denominators were extracted by risk group. Missing or unreported values were not imputed as zero.

### Risk of bias assessment

Risk of bias was assessed independently by two reviewers using the Newcastle-Ottawa Scale for cohort studies. The assessment focused on study selection, group comparability, outcome assessment, adequacy of follow-up, and potential bias from confounding, incomplete reporting, or cohort overlap. Disagreements were resolved by discussion or, when necessary, by consultation with a third reviewer.

### Statistical analysis

Quantitative synthesis was performed only when a given outcome–effect-measure combination included at least three independent estimates. Before pooling, estimates were required to be sufficiently comparable with respect to the study population, clinical context, outcome definition, assessment window, and effect measure. Odds ratios (ORs) and hazard ratios (HRs) were analyzed in separate models, and fixed-time or event-based outcomes were not combined with time-to-event outcomes. ICAHT, OS, and PFS were treated as the principal quantitative domains. Severe infection, early red blood cell transfusion, early platelet transfusion, severe CRS, and severe ICANS were considered supplementary exploratory domains when the same compatibility criteria were satisfied. Outcomes with fewer eligible estimates, incompatible definitions, or evident clinical heterogeneity were summarized narratively or in supplementary evidence tables.

For dichotomous or fixed-time outcomes, log odds ratios and corresponding standard errors were derived from reported estimates and 95% confidence intervals or, when appropriate, reconstructed from event counts. For time-to-event outcomes, log hazard ratios and standard errors were derived from reported hazard ratios and 95% confidence intervals. Adjusted and unadjusted estimates were not treated as interchangeable. When both were available for the same cohort and outcome, the adjusted estimate was preferred if the covariate model was clinically interpretable and broadly comparable with the other included estimates.

All pooled analyses used random-effects inverse-variance models because heterogeneity was expected across disease settings, CAR T-cell products, follow-up durations, outcome definitions, and adjustment strategies. Between-study variance was estimated by restricted maximum likelihood. Pooled effects are reported as ORs or HRs with 95% confidence intervals. Statistical heterogeneity was quantified with I² and tau² and assessed with the Cochran Q test. Leave-one-out analyses were performed for models containing more than three estimates when the resulting comparisons remained interpretable. In the ICAHT, OS, and PFS models, the omission of the sole relapsed/refractory multiple myeloma estimate left only lymphoma cohorts; these leave-one-out results were therefore examined as disease-restricted sensitivity analyses. Formal comparisons between disease subgroups were not undertaken because no second disease category contained enough estimates for pooling, and leukemia was not represented in these models. Age-group analyses were not possible because the compatible estimates were derived from adult cohorts and no separate pediatric estimate was available. Funnel plots, Egger tests, and meta-regression were not performed because of the small number of estimates within each outcome.

## Results

### Study selection

A total of 75 records were identified through database searches. After removal of 30 duplicates, 45 records were screened by title and abstract, of which 18 were excluded because they did not meet the eligibility criteria. Full texts were sought for the remaining 27 records; 23 were retrieved and assessed in full, whereas 4 could not be evaluated further because only bibliographic information was available and no full text could be retrieved. The study selection process is shown in **Supplementary Figure B1**.

### Study and exposure characteristics

The included studies were predominantly observational cohort studies, including retrospective multicenter analyses and registry-based cohorts (3, 5–26). Study populations included patients with large B-cell lymphoma, multiple myeloma, mantle cell lymphoma, B-cell acute lymphoblastic leukemia, and mixed hematologic malignancies. Where the treatment setting was reported, CAR T-cell therapy was administered for relapsed/refractory or otherwise previously treated disease. No included study specifically evaluated CAR T-cell therapy as first-line treatment. CAR T-cell products targeted either CD19 or BCMA. Most studies evaluated the original CAR-HEMATOTOX score by classifying patients into high- and low-risk groups. The original model-development study and subsequent validation cohorts provided the principal evidence for the quantitative analyses. Included study characteristics are summarized in **Table 1** and **Supplementary Table C1**.

**Table 1:** Included study characteristics.

First author year country	Study design	Cohort	Disease	CAR-T product	N	CAR-HT cutoff/group definition	High-risk/low-risk n	Median follow-up	Outcomes reported
Rejeski 2021 (3) Europe and United States	Multicenter retrospective model development and external validation	CAR-HEMATOTOX derivation/validation LBCL cohorts	Relapsed/refractory large B-cell lymphoma	Axi-cel or tisa-cel	258	CAR-HEMATOTOX score; high risk defined by score ≥2 vs low score 0-1/<2	Not separately captured	Cytopenia to day 60/160; response day 90	Severe/prolonged neutropenia, thrombocytopenia, anemia, aplastic recovery phenotype, hospitalization-related hematotoxicity, response
Rejeski 2023 International, six centers	International multicenter retrospective cohort	BCMA-directed CAR-T RRMM cohort	Relapsed/refractory multiple myeloma	Ide-cel 106; cilta-cel 7	113	HTlow score 0–1 vs HThigh score≥2	HTlow 63; HThigh 50	Median follow-up about 7.9 months	Prolonged severe neutropenia/aplastic recovery, severe infection, grade ≥3 ICANS, 1-year NRM, response, PFS, OS
de Boer 2025 (6) Netherlands	Population-based national validation cohort	Dutch CAR-T Tumorboard Consortium	Relapsed/refractory large B-cell lymphoma	Commercial CD19 CAR-T	245	Original HT cutoff ≥2 vs <2; recalibrated cutoff≥3 also evaluated	Original cutoff: HThigh 171, HTlow 74; recalibrated cutoff: HThigh 140, HTlow 105	Median follow-up 12 months	Clinically significant neutropenia, early/late grade ≥3 ICAHT, severe infection, PFS, OS, NRM
Nair 2025 (7) United States	Model development/validation	B-ALL ALL-HEMATOTOX cohort	B-cell acute lymphoblastic leukemia	CD19/28z, CD22/41BB, and CD19/CD22 dual-targeted CAR-T constructs	156	CAR-HT low <2 vs high ≥2; ALL-HT derivative score developed for B-ALL	CAR-HT high nearly 90%; ALL-HT high-risk 47%	Day 0–30 severe neutropenia/ICAHT window; shorter than original CAR-HT day 60 monitoring	ICAHT, severe/prolonged neutropenia, infection, response, OS; ALL-HT model performance
Cook 2026 (8) International/multicenter	Multicenter retrospective management cohort	BCMA-directed CAR-T hematologic toxicity cohort	Multiple myeloma	BCMA-directed CAR-T	224; CAR-HT available for 216	CAR-HT low <2 vs high ≥2	Low 86; high 130; missing 8	Median follow-up 17.2 months	Early ICAHT, anemia, thrombocytopenia, transfusion support, growth factors/stem-cell boosts, infection, CRS, PFS/OS
Liang 2025 (9) United States	Predictive model development/validation	Early ICAHT predictive model cohort	Hematologic malignancies	Commercial or investigational CAR-T	691	CAR-HEMATOTOX low vs high risk compared with eIPM Pre/eIPM Post; pre-lymphodepletion values used	Not fully extracted	Early ICAHT day 0-30; survival follow-up estimated by inverse Kaplan-Meier	Grade 3–4 early ICAHT, severe/prolonged ICAHT, model discrimination, OS
Valtis 2026 (10) United States	Consortium cohort with independent investigational cohort	ROCCA adult ALL brexu-cel; MSK 1928z validation	Adult acute lymphoblastic leukemia	Brexu-cel; investigational CD19 CAR-T (1928z) in validation cohort	199	CAR HEMATOTOX low vs high risk as reported	ROCCA: HTlow 43 (22%); HThigh 156 (78%)	12-month outcomes reported	Delayed neutrophil recovery, severe infection, MRD-negative CR/CRi, OS, EFS, NRM, relapse
Kampouri 2024 (11) United States	Prospective observational cohort	Fred Hutch/University of Washington CMV reactivation CARTx cohort	Hematologic malignancies; CMV-seropositive CARTx recipients	CD19/CD20- or BCMA-targeted CARTx; axi-cel, tisa-cel, liso-cel, brexu-cel, ide-cel, cilta-cel included	69	CAR-HEMATOTOX calculated as risk score/risk factor, not primary high-vs-low exposure	Not reported as high/low CAR-HT groups	Weekly CMV testing up to 12 weeks; median follow-up 81 days	CMV reactivation, CMV-specific cell-mediated immunity, CMV risk factors, infection subtype
Stella 2025 (12) Italy	Prospective observational registry analysis	CART-SIE	Large B-cell lymphoma	Commercial anti-CD19 CAR-T	746 infused; CAR-HEMATOTOX evaluable 389; simpleHT evaluable 560	CAR-HEMATOTOX high risk if score ≥2; simpleHT also evaluated	CAR-HT: low 138, high 251; simpleHT: low 465, high 95	Median follow-up 18 months; 1-year OS/PFS reported	Late grade ≥3 ICAHT, response at day 90, OS, PFS, secondary primary malignancy, survival stratification
Kaur 2026 (13) United States	Registry cohort	CIBMTR ide-cel cytopenia cohort	Multiple myeloma	Idecabtagene vicleucel	821 total; CAR-HEMATOTOX evaluable 168	High CAR-HEMATOTOX score≥2 evaluated for cytopenia association	Not fully extracted	Day 30 and day 100 cytopenia; survival outcomes	Day 30/day 100 cytopenias, hematologic recovery, infection/supportive outcomes, PFS, OS
Wiemers (14) 2025 Germany	Single-center observational cohort	BCMA-directed CAR-T splenomegaly cohort	Relapsed/refractory multiple myeloma	Ide-cel 35; cilta-cel 38	73	CAR-HEMATOTOX score assessed before lymphodepletion; score >1 considered high	Not fully extracted	Median follow-up: PFS 6.1 months; OS 8.2 months	Splenomegaly, thrombocytopenia, sBCMA/metabolic tumor volume, PFS, OS; CAR-HT comparator/covariate
Zhang (15) 2025 China	Prospective comparative score validation	Peking University People’s Hospital mixed hematologic malignancy CAR-T cohort	B-ALL, T-ALL/NHL, and multiple myeloma	B-ALL: CD19 or CD19/CD22; T-ALL/NHL: CD7; MM: BCMA; commercial and investigational products	119	CAR-HT, ALL-HT, and eIPM categories compared across diseases	CAR-HT high 80 (67.2%), low 39; ALL-HT high 56.3% of ALL/NHL patients	Follow-up collected through Aug 30, 2025; median follow-up varied by disease/risk group (about 5.9-14.2 months)	ICAHT grade/duration, prolonged neutropenia, hematologic recovery, OS, PFS, model performance
Airosa Pardal (16) 2025 Spain	Retrospective immune recovery cohort	Axi-cel aggressive B-cell lymphoma cohort	Aggressive B-cell lymphoma	Axicabtagene ciloleucel	76	CAR-HEMATOTOX high vs low at lymphodepletion as reported	Low 36 (47%); high 40 (53%)	12-month PFS/OS; immune recovery during first year	Immune recovery, CD4/NK/IgG recovery, infections, PFS, OS
Rejeski 2023 International, eight centers	International multicenter retrospective cohort	Brexu-cel mantle cell lymphoma cohort	Relapsed/refractory mantle cell lymphoma	Brexucabtagene autoleucel	103	HTlow score 0–1 vs HThigh score ≥2	HTlow 56; HThigh 47	1-year NRM and survival outcomes reported	Aplastic neutrophil recovery/prolonged cytopenia, severe infection, NRM, response, PFS, OS
Rejeski (18) 2022 International, six centers	International multicenter retrospective cohort	CD19 CAR-T LBCL infection cohort	Relapsed/refractory large B-cell lymphoma	Axi-cel or tisa-cel	248	HTlow score 0–1 vs HThigh score ≥2	HTlow 115 (46%); HThigh 133 (54%)	90-day infection; survival follow-up	Severe infection, bacterial infection, neutrophil recovery, hospitalization duration, PFS, OS, NRM
Srsen (19) 2026 Slovenia	Single-country real-world score evaluation	Adult DLBCL CAR-T Slovenia cohort	Diffuse large B-cell lymphoma	Commercial anti-CD19 CAR-T; tisagenlecleucel	14	CAR-HEMATOTOX assessed on day -5 and day 0 with EASIX-C and IBPS	Not fully extracted	Not fully extracted	Response/remission, severe cytopenias, CRS, ICANS, predictive score performance
Culbert (20) 2025 United States	Multicenter retrospective risk-factor cohort	NCI, MSKCC, and Seattle Children’s B-ALL infection cohort	B-cell acute lymphoblastic leukemia	CD19_28z, CD19_BB, and CD22_BB CAR-T constructs	350	ALL-HT derivative score primary; CAR-HT also evaluated for infection risk	ALL-HT low 179, high 171; CAR-HT high 232 (88% among evaluable patients)	Severe infection through day +60	Severe infection, mild infection, infection microbiology, prior infection, ICAHT/high-risk hematotoxicity, OS
Vic (21) 2024 France	National registry cohort	DESCAR-T	Large B-cell lymphoma	Commercial CD19 CAR-T	671; CAR-HEMATOTOX available for 611	CAR-HEMATOTOX score ≥2	High 383; low 228; missing 60	Median follow-up 18.2 months	Early RBC transfusion, early platelet transfusion, late transfusion, PFS, OS, NRM
Winkelmann (22) 2025 International, six sites	Multicenter observational model optimization/validation	CD19 CAR-T LBCL CAR-IMPI cohort	Large B-cell lymphoma	Axi-cel 285; liso-cel 70; tisa-cel 149	504	CAR-IMPI primary; exploratory combination with CAR-HEMATOTOX high defined as values ≥3 and low ≤2	Not fully extracted	Median follow-up 32.1 months overall	PFS, OS, CRS, ICANS, ICU admission, CAR-IMPI model performance; exploratory CAR-HT combination
Barone (23) 2026 Italy	Prospective registry analysis	CART-SIE NRM cohort	B-cell lymphomas receiving commercial CAR-T	Axi-cel 483; tisa-cel 323; brexu-cel 126	1132 enrolled; 932 outcome-evaluable	High CAR-HEMATOTOX as pre-infusion risk factor/covariate per Rejeski definition	High 556 (67%) among patients with CAR-HT data; low n not fully extracted	Median follow-up 17.8 months (IQR 6.3-25.4)	NRM, causes of NRM, infection-related mortality, CRS, ICANS, ICAHT, survival context
Nakamura (24) 2024 Japan	Single-center retrospective prediction-score cohort	Kyoto KyoTox A-score cohort	B-cell lymphoma after CAR-T	Tisa-cel 67; liso-cel 18; axi-cel 5	90 analyzed at 90 days after infusion	CAR-HEMATOTOX high vs low compared with KyoTox A-score	CAR-HEMATOTOX high 69 (76.7%); low 21 (23.3%)	Assessed through 90 days after infusion	Prolonged neutropenia, anemia, thrombocytopenia, prolonged cytopenia, KyoTox model performance
Rejeski 2023 Germany/Spain	Retrospective observational discovery study with multicenter validation cohort	CD19 CAR-T B-NHL HT10 cohort	Relapsed/refractory B-cell non-Hodgkin lymphoma	Discovery: axi-cel 23, tisa-cel 30, brexu-cel 9; validation CD19 CAR-T	Discovery n=62; validation n=125	HT10 combines baseline CAR-HEMATOTOX with day-of-fever procalcitonin; CAR-HT used as component	Not fully extracted	Early fever/infection day 0-30; validation at first fever	Early infection vs CRS, severe infection, bacterial/nonviral infection, HT10 AUC/model performance, proteomic signatures
Xu (26) 2026 China	Single-center retrospective nomogram construction/validation	RRMM CAR-T nutritional status/CONUT nomogram cohort	Relapsed/refractory multiple myeloma	CAR-T regimens for RRMM; BCMA/GPRC5D-related details not fully extracted	302	CAR-HEMATOTOX used as comparator model; not primary high/low clinical-effect exposure	Not fully extracted	Not fully extracted	Prolonged grade 3/4 neutropenia >28 days, prolonged hematological toxicity composite, CONUT nomogram performance, CAR-HT AUC comparator

Study characteristics, sample sizes, follow-up periods, CAR-HEMATOTOX definitions, and outcomes are presented as reported in the original publications. Sample sizes may differ across analyses because some scores or outcomes were available only in evaluable subsets.

Unless otherwise specified, the original CAR-HEMATOTOX classification defines a score of 0–1 as low risk and a score of ≥2 as high risk. Recalibrated cutoffs, derivative scores, and comparator models are reported according to the definitions used in the respective studies.

The outcomes listed represent those evaluated or reported in each study and do not necessarily indicate inclusion in the quantitative synthesis. Outcome-level eligibility and pooling decisions are detailed in **Supplementary Table C3**.

“Not reported” indicates that the relevant information was not provided in the source publication. “Not fully extracted” indicates that the available report did not permit reliable extraction of the complete numerical information required for this table; no values were inferred.ALL-HT, acute lymphoblastic leukemia HEMATOTOX score; AUC, area under the curve; B-ALL, B-cell acute lymphoblastic leukemia; BCMA, B-cell maturation antigen; CAR-HEMATOTOX or CAR-HT, chimeric antigen receptor HEMATOTOX score; CAR-IMPI, CAR T-cell International Metabolic Prognostic Index; CAR-T, chimeric antigen receptor T-cell therapy; CARTx, CAR T-cell therapy; CIBMTR, Center for International Blood and Marrow Transplant Research; CMV, cytomegalovirus; CONUT, Controlling Nutritional Status; CR, complete response; CRi, complete response with incomplete count recovery; CRS, cytokine release syndrome; DLBCL, diffuse large B-cell lymphoma; EASIX-C, Endothelial Activation and Stress Index-C; EFS, event-free survival; eIPM, early ICAHT prediction model; GPRC5D, G protein-coupled receptor class C group 5 member D; HThigh, high CAR-HEMATOTOX risk; HTlow, low CAR-HEMATOTOX risk; IBPS, Inflammation-Based Prognostic Score; ICAHT, immune effector cell-associated hematotoxicity; ICANS, immune effector cell-associated neurotoxicity syndrome; ICU, intensive care unit; IgG, immunoglobulin G; LBCL, large B-cell lymphoma; MCL, mantle cell lymphoma; MM, multiple myeloma; MRD, measurable residual disease; N, number of patients; NHL, non-Hodgkin lymphoma; NK, natural killer; NR, not reported; NRM, nonrelapse mortality; OS, overall survival; PFS, progression-free survival; R/R, relapsed or refractory; RBC, red blood cell; RRMM, relapsed or refractory multiple myeloma; sBCMA, soluble B-cell maturation antigen; T-ALL, T-cell acute lymphoblastic leukemia.

Several cohorts included patients who had undergone allo-HSCT before CAR T-cell therapy, with the largest proportions reported in B-cell acute lymphoblastic leukemia cohorts (7, 10, 20). Smaller numbers were reported in some lymphoma and myeloma cohorts (5, 6, 21). Post-CAR T-cell allo-HSCT was most relevant in acute lymphoblastic leukemia, where some responders proceeded to consolidative transplantation (7, 10). We found no report of patients being excluded solely because of prior allo-HSCT. Prior transplantation was included as a covariate in the multivariable OS model in one acute lymphoblastic leukemia study (10), whereas several other studies reported transplant history only as a baseline characteristic (5, 6, 11, 15, 17, 20, 21). The same acute lymphoblastic leukemia study incorporated post-CAR T-cell consolidation or maintenance therapy as a time-dependent covariate in the EFS analysis (10). Otherwise, time-to-event outcomes were generally censored at the last known follow-up rather than at subsequent allo-HSCT. Early hematotoxicity analyses in acute lymphoblastic leukemia were limited to day 30 in part because some patients proceeded rapidly to transplantation (7). Because transplant timing and analytical handling were inconsistently reported, no transplant-specific subgroup analysis or additional meta-analytic adjustment was possible; study-level details are provided in **Supplementary Table C2**.

### Outcome definitions and data availability

Outcome definitions varied across studies. Hematotoxicity outcomes included severe cytopenia, prolonged cytopenia, late grade ≥3 ICAHT, or related severe/prolonged hematotoxicity constructs. Infection outcomes included severe infection, any infection, bacterial infection, time-to-event infection, and model-performance endpoints. Compatible data were available for meta-analysis of ICAHT, OS, PFS, and severe infection. Additional exploratory pooling was possible for early red blood cell transfusion, early platelet transfusion, severe CRS, and severe ICANS. Exposure and outcome definitions are summarized in **Supplementary Table C3**.

### Quantitative synthesis

High CAR-HEMATOTOX risk was associated with higher odds of ICAHT (pooled OR, 5.58; 95% CI, 1.98 to 15.73; P = 0.001), with moderate heterogeneity (τ²=0.64; I²=60.8%). A high CAR-HEMATOTOX score was associated with worse OS (pooled HR, 3.36; 95% CI, 2.20 to 5.11; P<0.001), with no observed statistical heterogeneity (τ²=0.00; I²=0.0%). A high CAR-HEMATOTOX score was also associated with worse PFS (pooled HR, 2.74; 95% CI, 1.81 to 4.15; P<0.001), with low-to-moderate heterogeneity (τ²=0.06; I²=32.3%). These pooled analyses are shown in **Supplementary Figure B2**.

In the existing leave-one-out analyses, omission of the sole relapsed/refractory multiple myeloma estimate left only lymphoma cohorts. The direction of the associations was unchanged: high CAR-HEMATOTOX risk remained associated with hematotoxicity or ICAHT (OR, 4.99; 95% CI, 1.39 to 17.88; I²=69.6%), worse OS (HR, 3.34; 95% CI, 2.12 to 5.24; I²=0%), and worse PFS (HR, 2.56; 95% CI, 1.55 to 4.23; I²=36.9%). When the severe-infection analysis was restricted to lymphoma cohorts, high CAR-HEMATOTOX risk also remained associated with higher odds of severe infection (OR, 5.00; 95% CI, 2.29 to 10.93; I² = 54.2%). These results provide a lymphoma-restricted sensitivity assessment (**Supplementary Figure B3**).

A high CAR-HEMATOTOX score was associated with higher odds of severe infection (pooled OR, 5.18; 95% CI, 2.78 to 9.67; P<0.001), with moderate heterogeneity (τ²=0.22; I²=37.8%). Leave-one-out analyses did not materially alter the direction of the association. The severe-infection forest plot is shown in **Supplementary Figure B4**.

### Supplementary exploratory analyses

High-risk status was associated with higher odds of early red blood cell transfusion (pooled OR, 3.96; 95% CI, 1.26 to 12.45; P = 0.019; I²=87.8%) and early platelet transfusion (pooled OR, 6.21; 95% CI, 1.86 to 20.74; P = 0.003; I²=85.1%). Because heterogeneity was high, these findings should be interpreted cautiously.

High-risk status was not significantly associated with severe CRS (pooled OR, 1.71; 95% CI, 0.26 to 11.20; P = 0.575) or severe ICANS (pooled OR, 1.41; 95% CI, 0.81 to 2.45; P = 0.230). These analyses were exploratory because the evidence base was limited and the toxicity definitions were not identical. Supplementary forest plots are provided in **Supplementary Figures B5**–**B8**.

### Narrative synthesis and risk of bias

Findings from studies not included in the principal meta-analyses generally supported the same clinical pattern. Across studies using broader infection definitions, time-to-event analyses, or event-rate reporting, patients with a high CAR-HEMATOTOX score tended to experience a greater burden of infection, although the magnitude of the association varied across populations and assessment windows (18, 20, 25, 27). Available studies also suggested a higher risk of non-relapse or treatment-related mortality among high-risk patients, with infection frequently identified as an important contributor to these deaths (5, 17, 23). However, these observations were based on a small number of events and should be interpreted cautiously.

High-risk patients also generally required more supportive care, including red blood cell and platelet transfusions, and some studies reported longer hospitalization or delayed hematologic recovery (3, 5, 17, 21). In contrast, associations with treatment response were less consistent across disease settings and assessment time points. Overall, the narrative evidence suggests that a high CAR-HEMATOTOX score identifies patients with poorer hematopoietic reserve, greater infectious vulnerability, and less favorable clinical outcomes, while the evidence for treatment response and nonhematologic toxicities remains less consistent. Detailed outcome definitions and study-level decisions are provided in **Supplementary Table C3**.

Risk-of-bias assessment indicated that the evidence was mainly observational and limited by residual confounding, heterogeneous adjustment, incomplete reporting, variable follow-up, and possible cohort overlap. Overall, the pooled and narrative findings suggest that a high CAR-HEMATOTOX score is associated with hematotoxicity, severe infection, greater supportive-care requirements, and worse survival, but the strength of evidence remains limited by the small number of compatible studies and differences in outcome definitions. The results of the risk-of-bias assessment are detailed in **Supplementary Figure B9**.

## Discussion

This systematic review and meta-analysis suggests that a high CAR-HEMATOTOX score is associated with a higher risk of hematotoxicity, severe infection, and inferior survival after CAR T-cell therapy for hematologic malignancies. The association was most consistent for survival outcomes and severe infection, whereas the evidence for transfusion requirements, CRS, and ICANS was more limited. Overall, these findings support CAR-HEMATOTOX as a clinically relevant baseline risk-stratification marker, but not as a stand-alone tool for treatment selection.

The observed associations are biologically plausible. CAR-HEMATOTOX incorporates markers of baseline inflammation and hematopoietic reserve, both of which may reflect a patient’s capacity to recover after lymphodepletion and CAR T-cell therapy (1–3). Patients classified as high risk may therefore be more vulnerable to prolonged cytopenia, infectious complications, and greater supportive-care needs (1, 2, 5). At the same time, the score may also capture broader adverse prognostic features, including disease burden, prior treatment exposure, impaired marrow function, and systemic inflammation (3, 5, 6). For this reason, the present findings should be interpreted as prognostic associations rather than evidence that the score itself identifies a causal pathway.

The survival findings are clinically important but require cautious interpretation. High-risk patients had worse OS and PFS, which suggests that CAR-HEMATOTOX may reflect more than hematologic toxicity alone. However, available studies differed in disease setting, CAR T-cell product, follow-up duration, and covariate adjustment (3, 5, 6, 10, 17). Some estimates were adjusted, whereas others were unadjusted or incompletely reported. Prior allo-HSCT and post-CAR T-cell consolidative allo-HSCT were also handled inconsistently across studies (5–7, 10, 20). These differences limit the extent to which the pooled estimates can be interpreted as independent effects of CAR-HEMATOTOX status.

The available evidence also reflects the current use of CAR T-cell therapy mainly in relapsed/refractory or previously treated disease. Because no included study specifically examined first-line CAR T-cell therapy, and results were not reported consistently by number of prior treatment lines, we could not compare CAR-HEMATOTOX performance across treatment settings. Performance may differ earlier in the disease course, when patients may have less cumulative marrow injury and treatment-related inflammation. The score distribution, absolute event rates, and possibly the most informative cutoff may therefore differ from those observed in the cohorts included here. Validation in earlier-line populations will be needed before the present findings can be extended to that setting.

The infection findings are also relevant to clinical practice. Severe infection was more common among high-risk patients, consistent with the relationship between impaired marrow reserve, prolonged cytopenia, immune dysfunction, and infectious vulnerability after CAR T-cell therapy (1, 2, 18). In practice, a high score may help identify patients who require closer blood-count monitoring, earlier evaluation for infection, and more proactive supportive-care planning (1, 2). However, the studies used variable infection definitions and reporting windows, and not all infection-related outcomes could be combined. Future studies should distinguish severe infection, microbiologically documented infection, infection timing, and infection-related mortality using standardized definitions.

The exploratory findings for early red blood cell and platelet transfusion are directionally consistent with the hematotoxicity results, but heterogeneity was high. These analyses should therefore be viewed as supportive rather than definitive. In contrast, high CAR-HEMATOTOX status was not clearly associated with severe CRS or severe ICANS. This distinction is clinically plausible because CRS and ICANS are influenced by factors such as CAR T-cell product, tumor burden, immune activation kinetics, and toxicity management, whereas CAR-HEMATOTOX more directly reflects baseline inflammation and hematopoietic reserve (1–3).

This review differs from broader CAR T-cell adverse-event prediction reviews by focusing specifically on the original high- versus low-risk CAR-HEMATOTOX comparison and clinically interpretable outcomes (3, 4). Derivative scores and comparator models may be useful, but they should be evaluated separately unless a study is specifically designed to compare predictive performance across tools (7, 9, 15). Combining original and modified risk models without distinction would reduce clinical interpretability.

The main strengths of this review include a focused research question, separation of original CAR-HEMATOTOX from derivative tools, careful handling of potentially overlapping cohorts, and outcome-specific pooling. The limitations are also important. The number of compatible studies was small for several outcomes; definitions and time points varied; adjusted and unadjusted estimates were sometimes mixed; and residual cohort overlap could not be fully excluded. Reporting of allo-HSCT before and after CAR T-cell therapy was incomplete, and its analytical handling varied across studies. As individual patient data were unavailable, residual confounding related to transplant history could not be fully addressed in the meta-analysis. The existing leave-one-out results restricted to lymphoma cohorts were consistent with the main analyses, but they do not establish that CAR-HEMATOTOX performs similarly across malignancies. There were too few non-lymphoma estimates for a formal comparison, and leukemia was not represented in the principal pooled analyses. The compatible estimates were also drawn from adult cohorts, so the findings should not be assumed to apply to pediatric patients. In addition, the available evidence was observational, and small-study effects or publication bias could not be assessed reliably because of the limited number of studies per outcome.

Prospective work has begun to validate the prognostic performance of CAR-HEMATOTOX. In the prospective CART-SIE cohort, a high score was associated with late severe ICAHT, lower response rates, and inferior survival; however, that study did not evaluate whether management guided by the score improved clinical outcomes (12). The next step should therefore be to assess the clinical utility of CAR-HEMATOTOX rather than to repeat prognostic validation alone.

A high CAR-HEMATOTOX score should not, by itself, be used to exclude a patient from CAR T-cell therapy. Instead, it may identify patients who require a more intensive supportive-care plan. Before lymphodepletion, this could include evaluation of potentially reversible causes of cytopenia or inflammation, assessment for active infection, and advance planning for transfusion and hematologic support. After CAR T-cell infusion, high-risk patients may benefit from closer monitoring of blood counts and infectious complications and a lower threshold for diagnostic evaluation when fever or other signs of infection occur. Current expert recommendations allow consideration of early granulocyte colony-stimulating factor in patients at high risk for ICAHT and risk-adapted antibacterial or antifungal prophylaxis in patients with severe or prolonged neutropenia (1, 2). Retrospective evidence has also suggested that antibacterial prophylaxis may reduce severe bacterial infections in patients classified as high risk (18). These measures should nevertheless be individualized because their benefits, adverse effects, and optimal timing have not been established in CAR-HEMATOTOX-guided prospective trials.

Future studies should compare a prespecified CAR-HEMATOTOX-guided care pathway with standard supportive care. Relevant outcomes should include the duration of severe neutropenia, febrile neutropenia, grade ≥3 infection, transfusion requirements, hospitalization, intensive-care use, antimicrobial exposure, treatment-related mortality, quality of life, and healthcare utilization. These studies should also determine whether intensified supportive care reduces complications without increasing CRS or ICANS, delaying CAR T-cell therapy, or compromising disease control. Evaluation across disease groups and CAR T-cell products, together with assessment of clinical utility and cost-effectiveness, would help establish whether use of the score improves patient management rather than simply identifying patients with a worse prognosis.

## Conclusion

In this systematic review and meta-analysis, a high CAR-HEMATOTOX score was associated with greater risks of severe or prolonged hematotoxicity and severe infection, as well as inferior OS and PFS after CAR T-cell therapy for hematologic malignancies. Associations with early red blood cell and platelet transfusion requirements were less robust because of substantial between-study heterogeneity, whereas no consistent association was observed with severe CRS or severe ICANS. These findings support CAR-HEMATOTOX as a baseline prognostic marker for monitoring and supportive-care planning, rather than as a stand-alone treatment-decision tool. Prospective studies using standardized ICAHT, infection, and survival endpoints are needed to clarify its clinical utility.

## Data Availability

The original contributions presented in the study are included in the article/**Supplementary Material**. Further inquiries can be directed to the corresponding author.
