# Supplementary material for: CAR-HEMATOTOX score as a predictor of hematotoxicity, infection, and survival after CAR T-cell therapy in hematologic malignancies: a systematic review and meta-analysis
Source: Front Oncol. 2026 Sep 1;16:1928438. doi: 10.3389/fonc.2026.1928438 (PMC13574628; doi:10.3389/fonc.2026.1928438)
Supplement: Supplementary file 1 [file DataSheet1.pdf]

Supplementary Information(SI)

Article title: CAR-HEMATOTOX score as a predictor of hematotoxicity, infection, and survival after CAR T-cell therapy in hematologic malignancies: a systematic review and meta-analysis

Journal name: Frontiers in Oncology

Author names: Juncheng Wei, Ziyu Chen, Mengfei Li, Miao Jiang*

Affiliation and e-mail address of the corresponding author: College of Life Sciences, Beijing University of Chinese Medicine; 700589@bucm.edu.cn

This file includes:

A. Supplementary notes

Notes A1 to A2.

B. Supplementary figures

Figures B1 to B9.

C. Supplementary tables

Tables C1 to C3.

A. Supplementary notes

Notes A1: PRISMA checklist

Section and Topic	Item #	Checklist item	Location where item is reported
**TITLE**			
Title	1	Identify the report as a systematic review.	1
**ABSTRACT**			
Abstract	2	See the PRISMA 2020 for Abstracts checklist.	1-2
**INTRODUCTION**			
Rationale	3	Describe the rationale for the review in the context of existing knowledge.	2
Objectives	4	Provide an explicit statement of the objective(s) or question(s) the review addresses.	2
**METHODS**			
Eligibility criteria	5	Specify the inclusion and exclusion criteria for the review and how studies were grouped for the syntheses.	3-4
Information sources	6	Specify all databases, registers, websites, organisations, reference lists and other sources searched or consulted to identify studies. Specify the date when each source was last searched or consulted.	3
Search strategy	7	Present the full search strategies for all databases, registers and websites, including any filters and limits used.	3
Selection process	8	Specify the methods used to decide whether a study met the inclusion criteria of the review, including how many reviewers screened each record and each report retrieved, whether they worked independently, and if applicable, details of automation tools used in the process.	4
Data collection process	9	Specify the methods used to collect data from reports, including how many reviewers collected data from each report, whether they worked independently, any processes for obtaining or confirming data from study investigators, and if applicable, details of automation tools used in the process.	4
Data items	10a	List and define all outcomes for which data were sought. Specify whether all results that were compatible with each outcome domain in each study were sought (e.g. for all measures, time points, analyses), and if not, the methods used to decide which results to collect.	3-4
	10b	List and define all other variables for which data were sought (e.g. participant and intervention characteristics, funding sources). Describe any assumptions made about any missing or unclear information.	4
Study risk of bias assessment	11	Specify the methods used to assess risk of bias in the included studies, including details of the tool(s) used, how many reviewers assessed each study and whether they worked independently, and if applicable, details of automation tools used in the process.	4
Effect measures	12	Specify for each outcome the effect measure(s) (e.g. risk ratio, mean difference) used in the synthesis or presentation of results.	4-5
Synthesis methods	13a	Describe the processes used to decide which studies were eligible for each synthesis (e.g. tabulating the study intervention characteristics and comparing against the planned groups for each synthesis (item #5)).	3-5
	13b	Describe any methods required to prepare the data for presentation or synthesis, such as handling of missing summary statistics, or data conversions.	5

Section and Topic	Item #	Checklist item	Location where item is reported
	13c	Describe any methods used to tabulate or visually display results of individual studies and syntheses.	6-8
	13d	Describe any methods used to synthesize results and provide a rationale for the choice(s). If meta-analysis was performed, describe the model(s), method(s) to identify the presence and extent of statistical heterogeneity, and software package(s) used.	4-5
	13e	Describe any methods used to explore possible causes of heterogeneity among study results (e.g. subgroup analysis, meta-regression).	5
	13f	Describe any sensitivity analyses conducted to assess robustness of the synthesized results.	5,7
Reporting bias assessment	14	Describe any methods used to assess risk of bias due to missing results in a synthesis (arising from reporting biases).	5
Certainty assessment	15	Describe any methods used to assess certainty (or confidence) in the body of evidence for an outcome.	8,10
**RESULTS**			
Study selection	16a	Describe the results of the search and selection process, from the number of records identified in the search to the number of studies included in the review, ideally using a flow diagram.	5
	16b	Cite studies that might appear to meet the inclusion criteria, but which were excluded, and explain why they were excluded.	5
Study characteristics	17	Cite each included study and present its characteristics.	6
Risk of bias in studies	18	Present assessments of risk of bias for each included study.	8
Results of individual studies	19	For all outcomes, present, for each study: (a) summary statistics for each group (where appropriate) and (b) an effect estimate and its precision (e.g. confidence/credible interval), ideally using structured tables or plots.	6-8
Results of syntheses	20a	For each synthesis, briefly summarise the characteristics and risk of bias among contributing studies.	6-8
	20b	Present results of all statistical syntheses conducted. If meta-analysis was done, present for each the summary estimate and its precision (e.g. confidence/credible interval) and measures of statistical heterogeneity. If comparing groups, describe the direction of the effect.	6-7
	20c	Present results of all investigations of possible causes of heterogeneity among study results.	5,7
	20d	Present results of all sensitivity analyses conducted to assess the robustness of the synthesized results.	7
Reporting biases	21	Present assessments of risk of bias due to missing results (arising from reporting biases) for each synthesis assessed.	5,10
Certainty of evidence	22	Present assessments of certainty (or confidence) in the body of evidence for each outcome assessed.	8,10
**DISCUSSION**			
Discussion	23a	Provide a general interpretation of the results in the context of other evidence.	8-10
	23b	Discuss any limitations of the evidence included in the review.	8-10

Section and Topic	Item #	Checklist item	Location where item is reported
	23c	Discuss any limitations of the review processes used.	10
	23d	Discuss implications of the results for practice, policy, and future research.	9-10
**OTHER INFORMATION**			
Registration and protocol	24a	Provide registration information for the review, including register name and registration number, or state that the review was not registered.	3
	24b	Indicate where the review protocol can be accessed, or state that a protocol was not prepared.	3
	24c	Describe and explain any amendments to information provided at registration or in the protocol.	3
Support	25	Describe sources of financial or non-financial support for the review, and the role of the funders or sponsors in the review.	11
Competing interests	26	Declare any competing interests of review authors.	11
Availability of data, code and other materials	27	Report which of the following are publicly available and where they can be found: template data collection forms; data extracted from included studies; data used for all analyses; analytic code; any other materials used in the review.	6

Notes A2: Full database strategies

PubMed, Web of Science, and the Cochrane Library were searched on 19 June 2026.

PubMed

(("CAR-HEMATOTOX"[Title/Abstract] OR "CAR HEMATOTOX"[Title/Abstract] OR "CAR-HT"[Title/Abstract] OR "CAR HT"[Title/Abstract] OR "ALL-Hematotox"[Title/Abstract] OR "ALL Hematotox"[Title/Abstract] OR "ALL-HT"[Title/Abstract] OR "ALL HT"[Title/Abstract] OR "early ICAHT prediction model"[Title/Abstract] OR eIPM[Title/Abstract])

AND

("Receptors, Chimeric Antigen"[MeSH Terms] OR "Immunotherapy, Adoptive"[MeSH Terms] OR "CAR T"[Title/Abstract] OR "CAR-T"[Title/Abstract] OR "CAR T-cell"[Title/Abstract] OR "CAR-T cell"[Title/Abstract] OR "CAR T cells"[Title/Abstract] OR "CAR-T cells"[Title/Abstract] OR "chimeric antigen receptor T"[Title/Abstract] OR "chimeric antigen receptor T-cell"[Title/Abstract] OR "chimeric antigen receptor T cells"[Title/Abstract] OR CART[Title/Abstract] OR axicabtagene[Title/Abstract] OR tisagenlecleucel[Title/Abstract] OR lisocabtagene[Title/Abstract] OR brexucabtagene[Title/Abstract] OR idecabtagene[Title/Abstract] OR ciltacabtagene[Title/Abstract] OR "axi-cel"[Title/Abstract] OR "tisa-cel"[Title/Abstract] OR "liso-cel"[Title/Abstract] OR "brexu-cel"[Title/Abstract] OR "ide-cel"[Title/Abstract] OR "cilta-cel"[Title/Abstract])

AND

("Hematologic Neoplasms"[MeSH Terms] OR "Leukemia"[MeSH Terms] OR "Lymphoma"[MeSH Terms] OR "Multiple Myeloma"[MeSH Terms] OR "Cytopenia"[MeSH Terms] OR "Neutropenia"[MeSH Terms] OR "Thrombocytopenia"[MeSH Terms] OR "Anemia"[MeSH Terms] OR "Infections"[MeSH Terms] OR "Survival"[MeSH Terms] OR "Mortality"[MeSH Terms] OR "Hospitalization"[MeSH Terms] OR "Cytokine Release Syndrome"[MeSH Terms] OR "Neurotoxicity Syndromes"[MeSH Terms] OR hematologic malignan*[Title/Abstract] OR haematologic malignan*[Title/Abstract] OR hematological malignan*[Title/Abstract] OR haematological malignan*[Title/Abstract] OR leukemia*[Title/Abstract] OR leukaemia*[Title/Abstract] OR lymphoma*[Title/Abstract] OR myeloma*[Title/Abstract] OR hematotoxic*[Title/Abstract] OR haematotoxic*[Title/Abstract] OR cytopenia*[Title/Abstract] OR neutropenia*[Title/Abstract] OR thrombocytopenia*[Title/Abstract] OR anemia[Title/Abstract] OR anaemia[Title/Abstract] OR ICAHT[Title/Abstract] OR infection*[Title/Abstract] OR survival[Title/Abstract] OR mortality[Title/Abstract] OR "overall survival"[Title/Abstract] OR "progression-free survival"[Title/Abstract] OR "event-free survival"[Title/Abstract] OR "non-relapse mortality"[Title/Abstract] OR "treatment-related mortality"[Title/Abstract] OR hospitalization[Title/Abstract] OR hospitalisation[Title/Abstract] OR "length of stay"[Title/Abstract] OR CRS[Title/Abstract] OR ICANS[Title/Abstract]))

Web of Science

TS=(("CAR-HEMATOTOX" OR "CAR HEMATOTOX" OR "CAR-HT" OR "CAR HT" OR "ALL-Hematotox" OR "ALL Hematotox" OR "ALL-HT" OR "ALL HT" OR "early ICAHT prediction model" OR eIPM)

AND

("CAR T" OR "CAR-T" OR "CAR T-cell" OR "CAR-T cell" OR "CAR T cells" OR "CAR-T cells" OR "chimeric antigen receptor T" OR "chimeric antigen receptor T-cell" OR "chimeric antigen receptor T cells" OR CART OR axicabtagene OR tisagenlecleucel OR lisocabtagene OR brexucabtagene OR idecabtagene OR ciltacabtagene OR "axi-cel" OR "tisa-cel" OR "liso-cel" OR "brexu-cel" OR "ide-cel" OR "cilta-cel")

AND

((hematologic NEAR/1 malignan*) OR (haematologic NEAR/1 malignan*) OR (hematological NEAR/1 malignan*) OR (haematological NEAR/1 malignan*) OR leukemi* OR leukaemi* OR lymphoma* OR myeloma* OR hematotoxic* OR

haematotoxic* OR cytopenia* OR neutropenia* OR thrombocytopenia* OR anemia OR anaemia OR ICAHT OR infection* OR survival OR mortality OR "overall survival" OR "progression-free survival" OR "event-free survival" OR "non-relapse mortality" OR "treatment-related mortality" OR hospitalization OR hospitalisation OR "length of stay" OR CRS OR ICANS))

Cochrane Library

#1 ("CAR-HEMATOTOX" OR "CAR HEMATOTOX" OR "CAR-HT" OR "CAR HT" OR "ALL-Hematotox" OR "ALL Hematotox" OR "ALL-HT" OR "ALL HT" OR "early ICAHT prediction model" OR eIPM):ti,ab,kw

#2 ([mh "Receptors, Chimeric Antigen"] OR [mh "Immunotherapy, Adoptive"] OR ("CAR T" OR "CAR-T" OR "CAR T-cell" OR "CAR-T cell" OR "CAR T cells" OR "CAR-T cells" OR "chimeric antigen receptor T" OR "chimeric antigen receptor T-cell" OR "chimeric antigen receptor T cells" OR CART OR axicabtagene OR tisagenlecleucel OR lisocabtagene OR brexucabtagene OR idecabtagene OR ciltacabtagene OR "axi-cel" OR "tisa-cel" OR "liso-cel" OR "brexu-cel" OR "ide-cel" OR "cilta-cel"):ti,ab,kw)

#3 ([mh "Hematologic Neoplasms"] OR [mh "Leukemia"] OR [mh "Lymphoma"] OR [mh "Multiple Myeloma"] OR [mh "Cytopenia"] OR [mh "Neutropenia"] OR [mh "Thrombocytopenia"] OR [mh "Anemia"] OR [mh "Infections"] OR [mh "Survival"] OR [mh "Mortality"] OR [mh "Hospitalization"] OR [mh "Cytokine Release Syndrome"] OR [mh "Neurotoxicity Syndromes"] OR (hematologic NEXT malignan* OR haematologic NEXT malignan* OR hematological NEXT malignan* OR haematological NEXT malignan* OR leukemi* OR leukaemi* OR lymphoma* OR myeloma* OR hematotoxic* OR haematotoxic* OR cytopenia* OR neutropenia* OR thrombocytopenia* OR anemia OR anaemia OR ICAHT OR infection* OR survival OR mortality OR "overall survival" OR "progression-free survival" OR "event-free survival" OR "non-relapse mortality" OR "treatment-related mortality" OR hospitalization OR hospitalisation OR "length of stay" OR CRS OR ICANS):ti,ab,kw)

#4 #1 AND #2 AND #3

B. Supplementary figures

Fig. B1. PRISMA Flow Diagram.

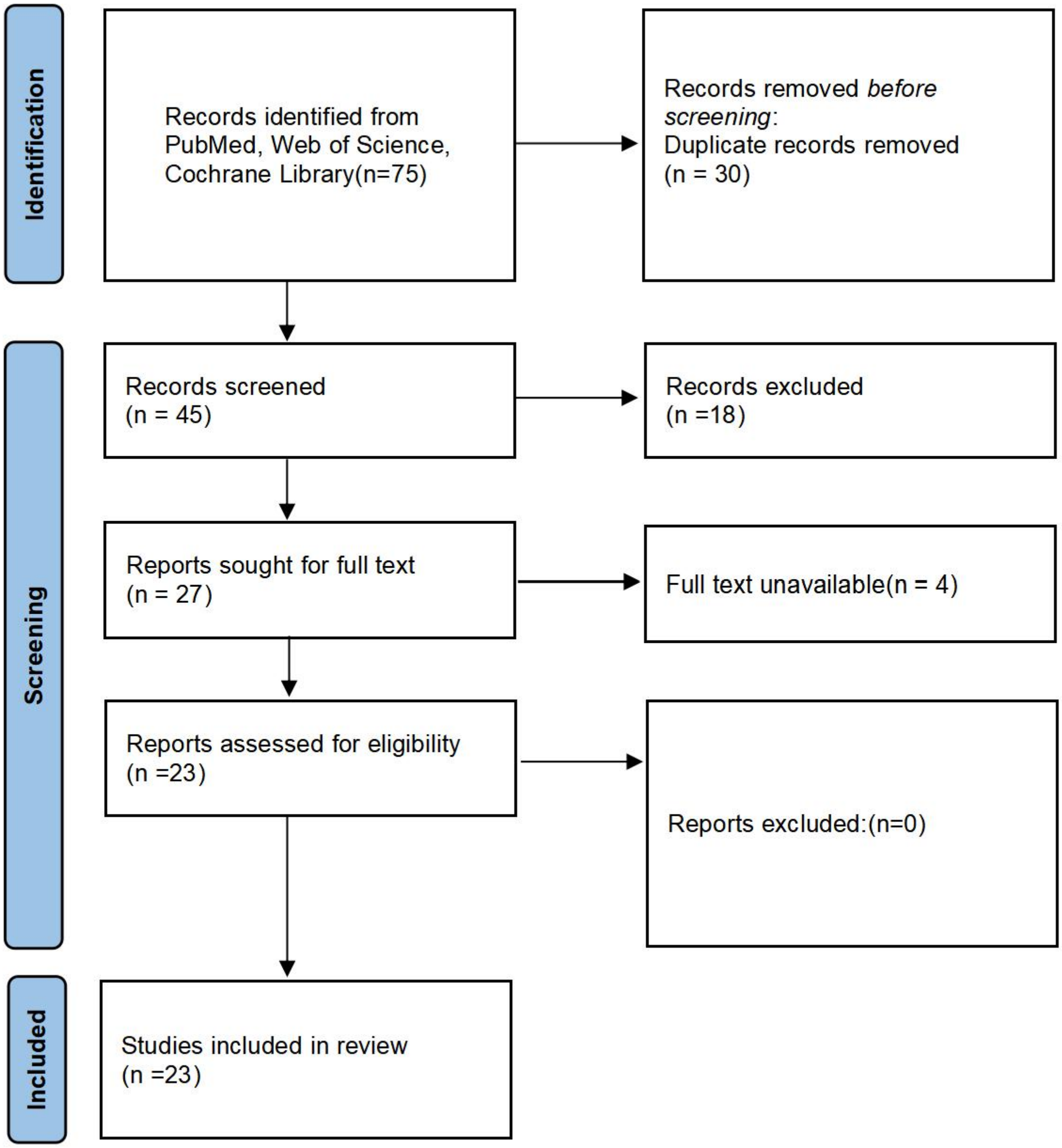

Fig. B2. Pooled associations of high versus low CAR-HEMATOTOX risk groups with hematotoxicity and survival outcomes.

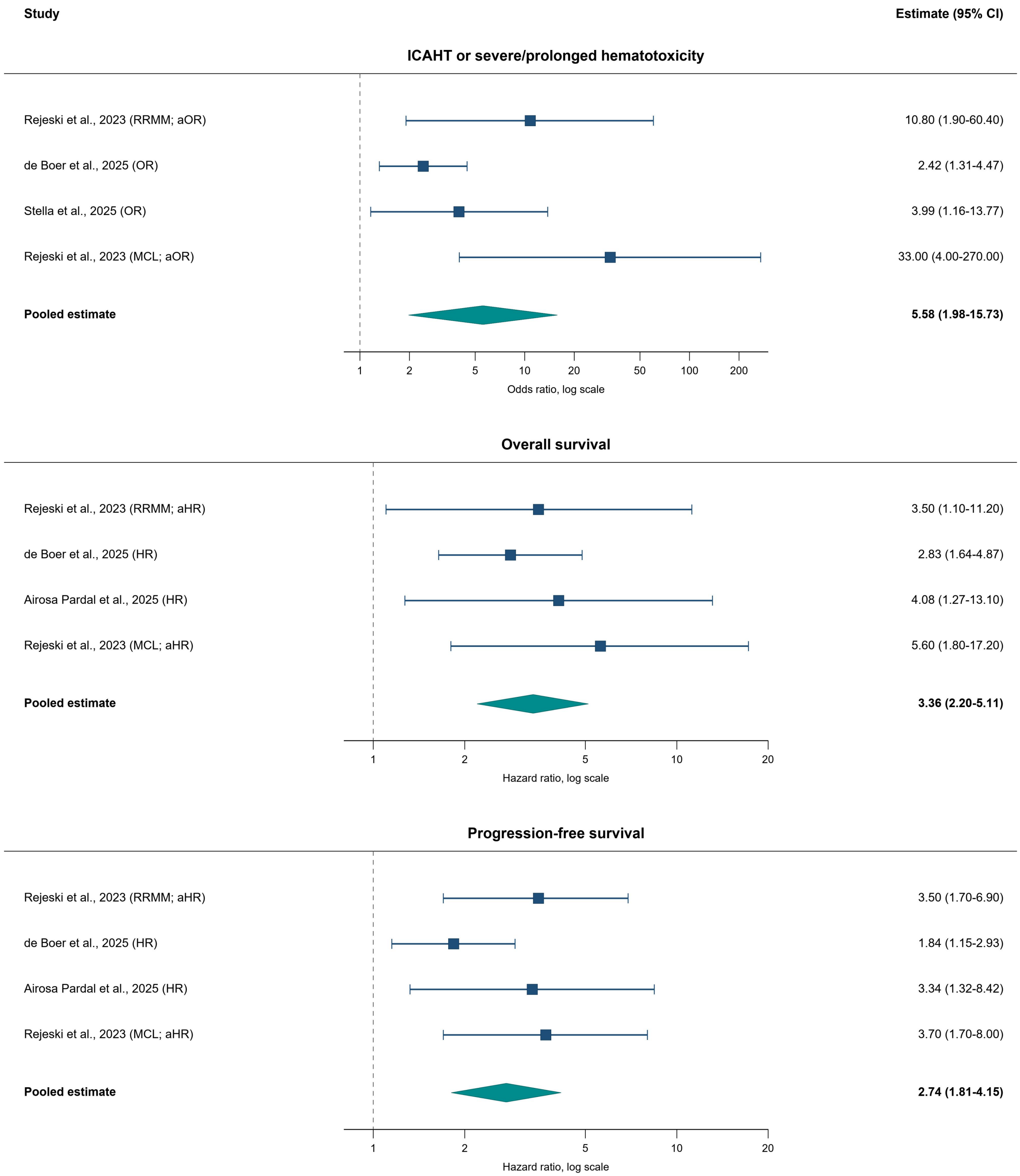

Odds ratios and hazard ratios greater than 1 indicate a higher risk of the corresponding adverse outcome in the high CAR-HEMATOTOX group than in the low CAR-HEMATOTOX group.

Fig. B3. Lymphoma-restricted sensitivity analyses of the associations of high versus low CAR-HEMATOTOX risk groups with hematotoxicity/ICAHT, overall survival, progression-free survival, and severe infection.

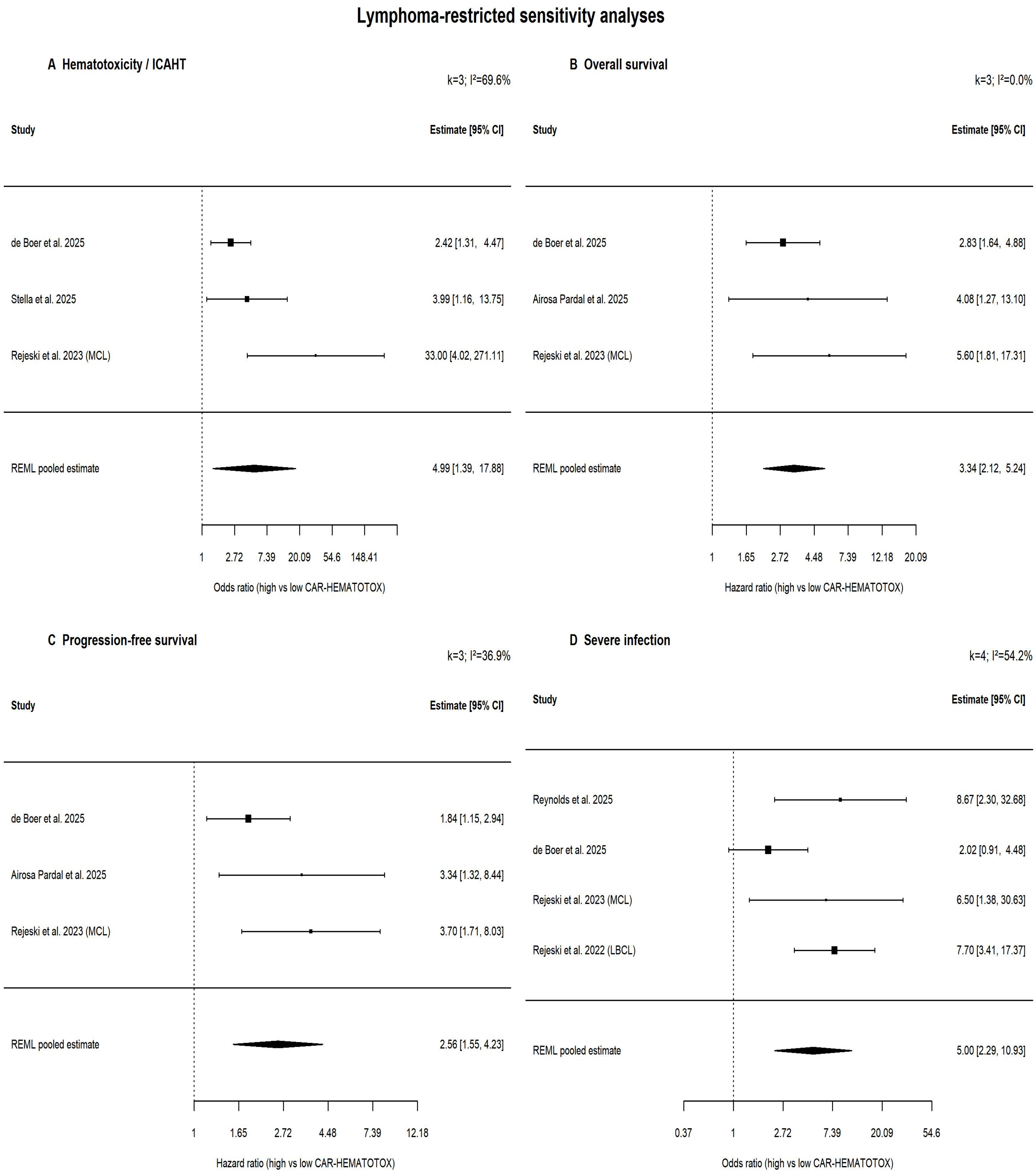

The sole relapsed/refractory multiple myeloma estimate was excluded, leaving lymphoma cohorts only. Formal comparisons between lymphoma and leukemia were not possible because leukemia was not represented among the compatible estimates included in these models.

Fig. B4. Severe infection: high versus low CAR-HEMATOTOX risk groups.

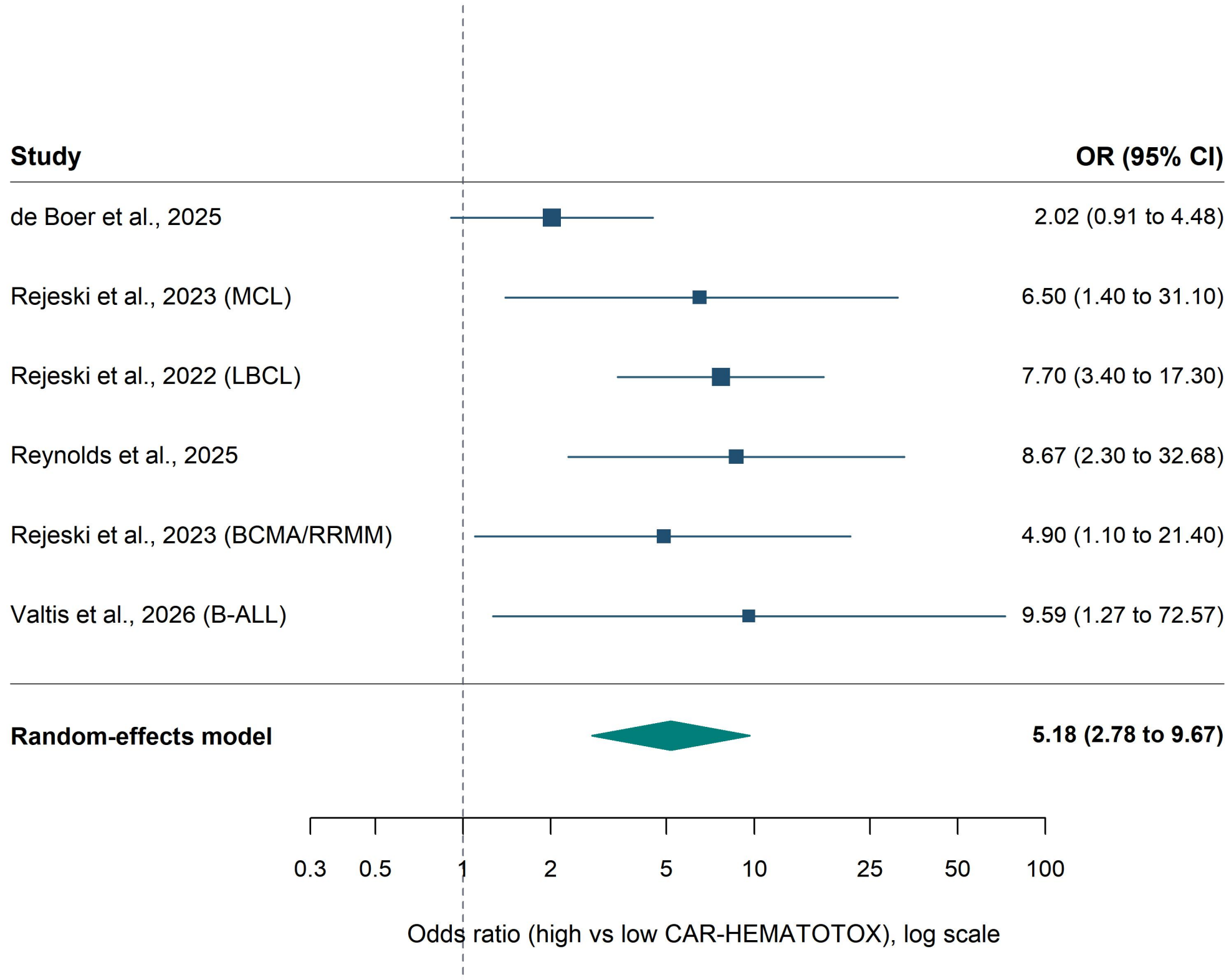

Odds ratios greater than 1 indicate a higher risk of severe infection in the high CAR-HEMATOTOX group than in the low CAR-HEMATOTOX group.

Fig. B5. Early red blood cell transfusion: high versus low CAR-HEMATOTOX risk groups.

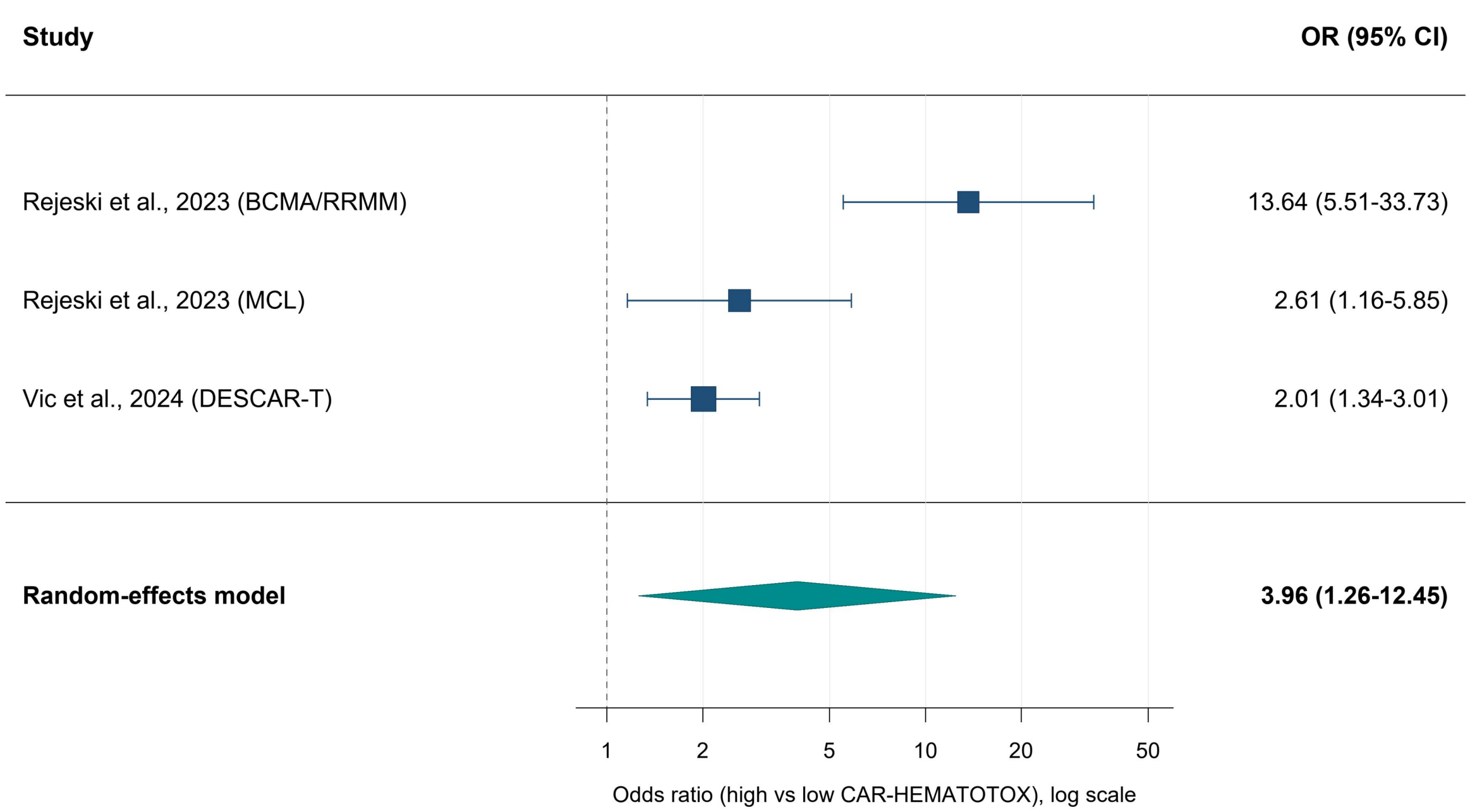

Odds ratios greater than 1 indicate a higher likelihood of early red blood cell transfusion in the high CAR-HEMATOTOX group than in the low CAR-HEMATOTOX group.

Fig. B6. Early platelet transfusion: high versus low CAR-HEMATOTOX risk groups.

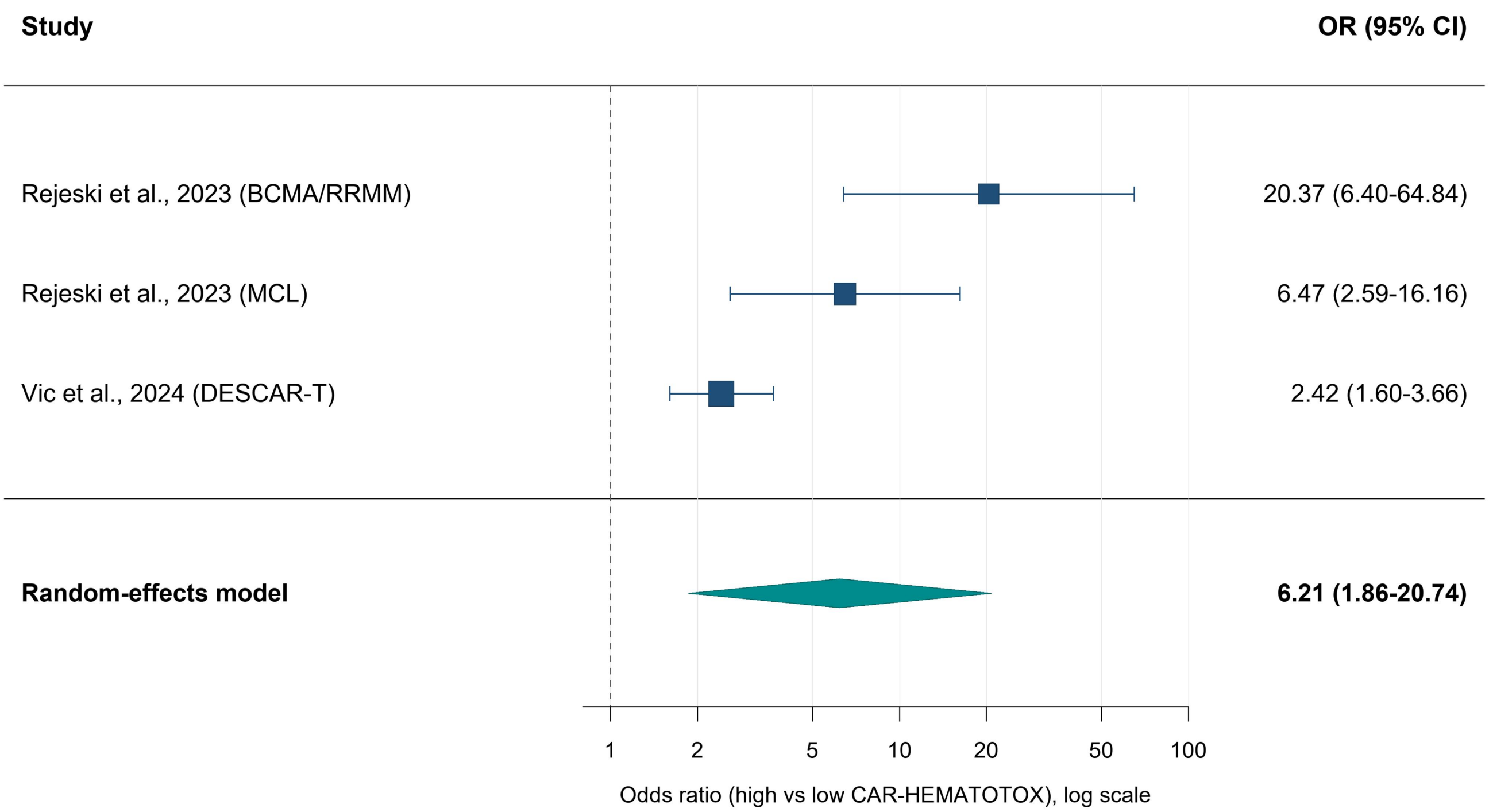

Odds ratios greater than 1 indicate a higher likelihood of early platelet transfusion in the high CAR-HEMATOTOX group than in the low CAR-HEMATOTOX group.

Fig. B7. Severe CRS: high versus low CAR-HEMATOTOX risk groups.

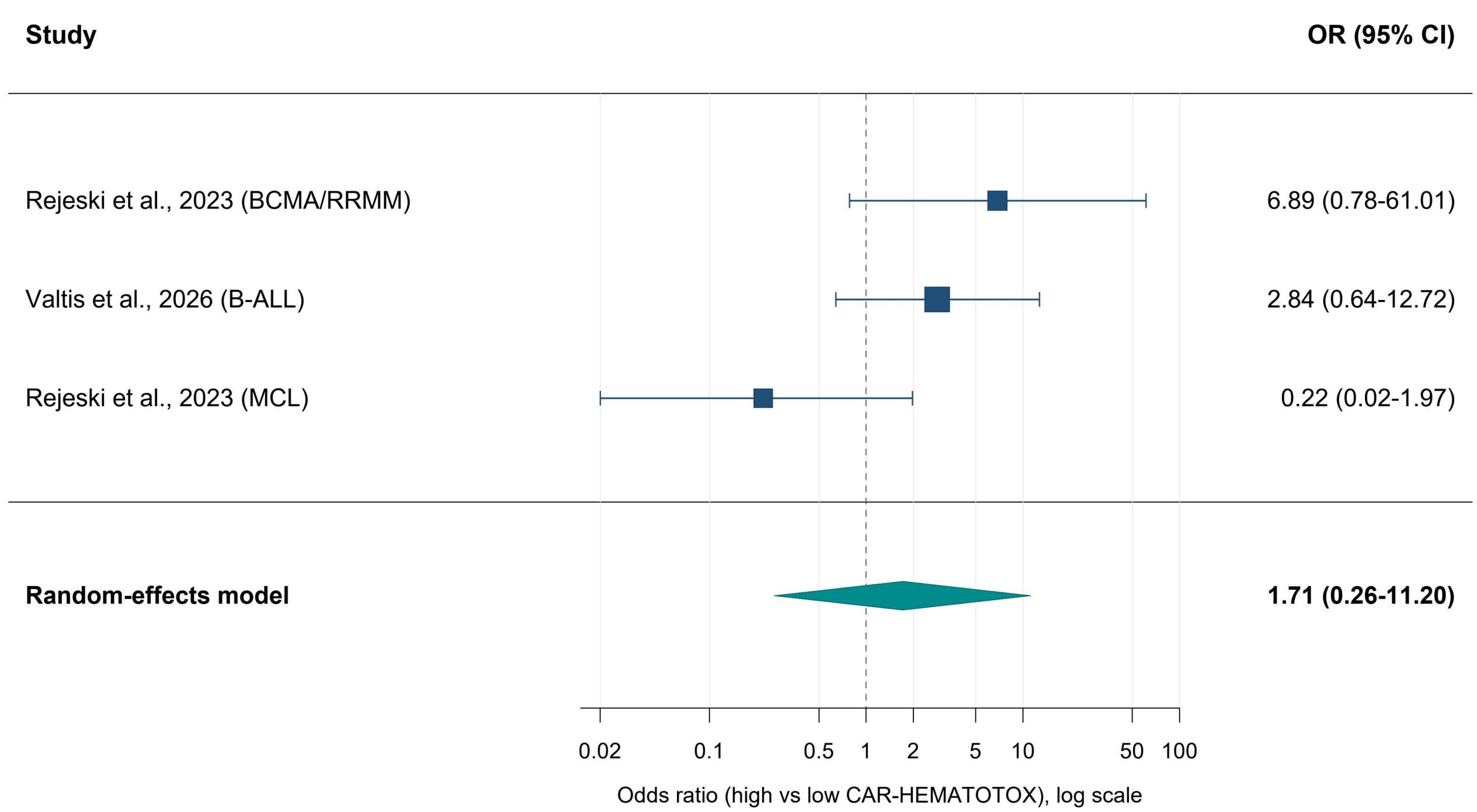

Odds ratios greater than 1 indicate a higher risk of severe cytokine release syndrome in the high CAR-HEMATOTOX group than in the low CAR-HEMATOTOX group.

Fig. B8. Severe ICANS: high versus low CAR-HEMATOTOX risk groups.

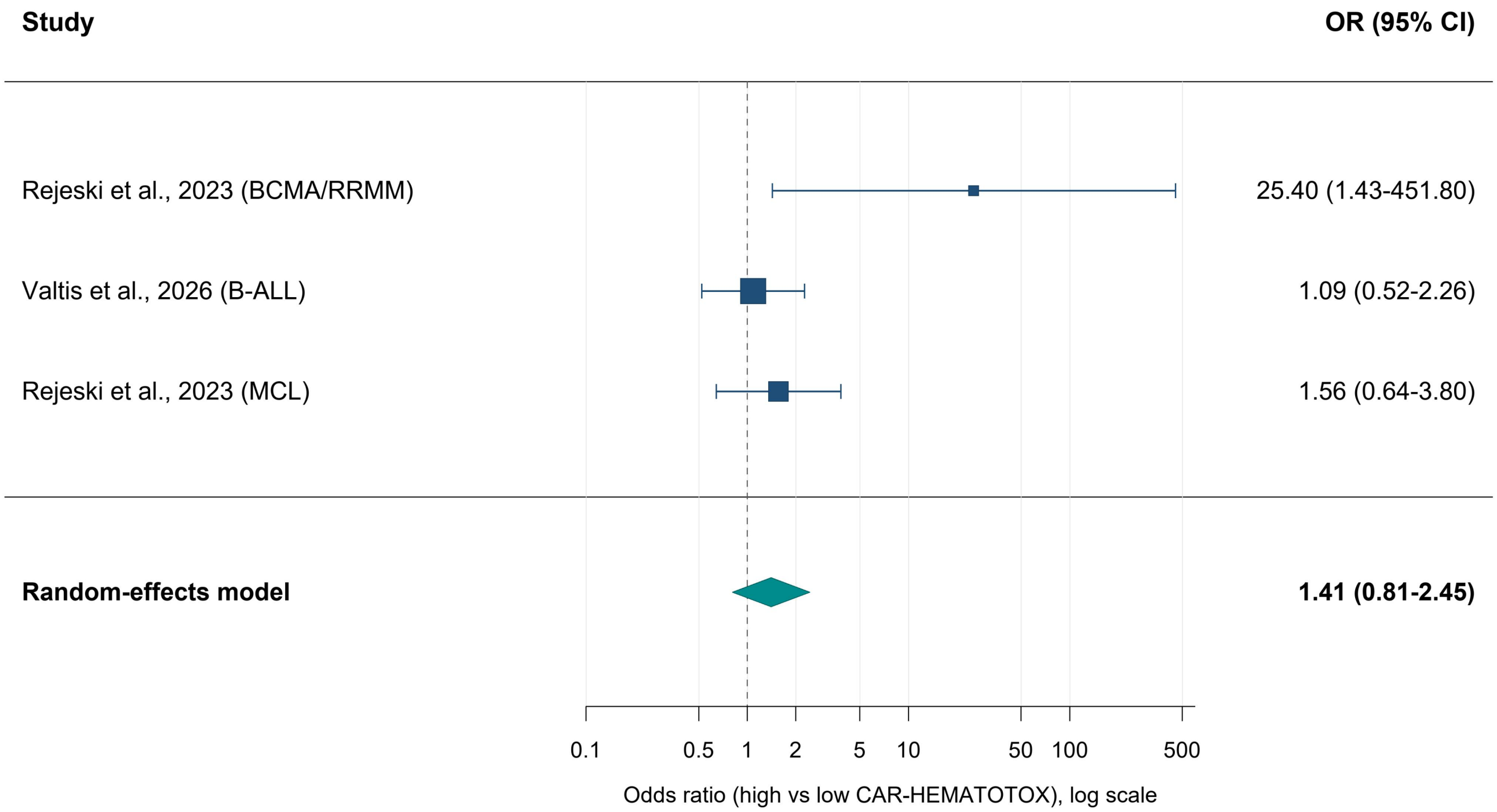

Odds ratios greater than 1 indicate a higher risk of severe immune effector cell-associated neurotoxicity syndrome in the high CAR-HEMATOTOX group than in the low CAR-HEMATOTOX group.

Fig. B9. Provisional risk-of-bias assessment of the included evidence.

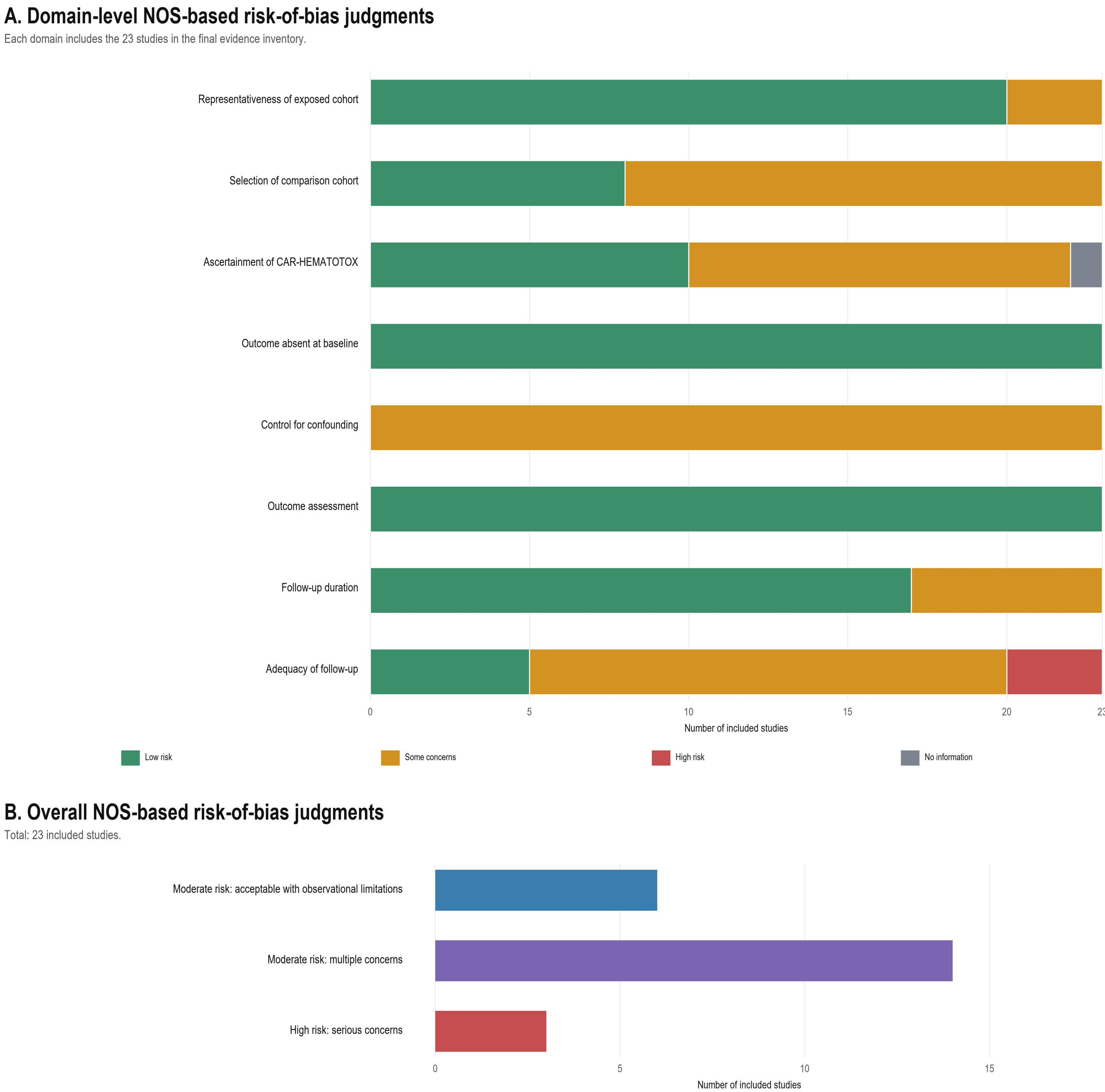

(A) Distribution of domain-level Newcastle–Ottawa Scale (NOS)-based judgments across the included studies. Each study contributed one judgment per domain.

(B) Distribution of the overall NOS-based risk-of-bias judgments.

C. Supplementary tables

Table C1. Included study characteristics.

Author Year Country	Study design	Cohort	Disease	CAR-T product	N	CAR-HT cutoff/group definition	High-risk/Low-risk n	Median follow-up	Outcomes reported
**Rejeski 2021 Europe and United States**	Multicenter retrospective model development and external validation	CAR-HEMATOTOX derivation/validation LBCL cohorts	Relapsed/refractory large B-cell lymphoma	Axi-cel or tisa-cel	258	CAR-HEMATOTOX score; high risk defined by score ⩾2 vs low score 0-1/<2	Not separately captured	Cytopenia to day 60/160; response day 90	Severe/prolonged neutropenia, thrombocytopenia, anemia, aplastic recovery phenotype, hospitalization-related hematotoxicity, response
**Rejeski 2023 International, six centers**	International multicenter retrospective cohort	BCMA-directed CAR-T RRMM cohort	Relapsed/refractory multiple myeloma	Ide-cel 106; cilta-cel 7	113	HTlow score 0-1 vs HThigh score ⩾2	HTlow 63; HThigh 50	Median follow-up about 7.9 months	Prolonged severe neutropenia/aplastic recovery, severe infection, grade ⩾3 ICANS, 1-year NRM, response, PFS, OS
**de Boer 2025 Netherlands**	Population-based national validation cohort	Dutch CAR-T Tumorboard Consortium	Relapsed/refractory large B-cell lymphoma	Commercial CD19 CAR-T	245	Original HT cutoff ⩾2 vs <2; recalibrated cutoff⩾3 also evaluated	Original cutoff: HThigh 171, HTlow 74; recalibrated cutoff: HThigh 140, HTlow 105	Median follow-up 12 months	Clinically significant neutropenia, early/late grade ⩾3 ICAHT, severe infection, PFS, OS, NRM
**Nair 2025 United States**	Model development/validation	B-ALL ALL-HEMATOTOX cohort	B-cell acute lymphoblastic leukemia	CD19/28z, CD22/41BB, and CD19/CD22 dual-targeted CAR-T constructs	156	CAR-HT low <2 vs high ⩾ 2; ALL-HT derivative score developed for B-ALL	CAR-HT high nearly 90%; ALL-HT high-risk 47%	Day 0-30 severe neutropenia/ICAHT window; shorter than original CAR-HT day 60 monitoring	ICAHT, severe/prolonged neutropenia, infection, response, OS; ALL-HT model performance
**Cook 2026 International/multicenter**	Multicenter retrospective management cohort	BCMA-directed CAR-T hematologic toxicity cohort	Multiple myeloma	BCMA-directed CAR-T	224; CAR-HT available for 216	CAR-HT low <2 vs high ⩾ 2	Low 86; high 130; missing 8	Median follow-up 17.2 months	Early ICAHT, anemia, thrombocytopenia, transfusion support, growth

Author<br>Year<br>Country	Study design	Cohort	Disease	CAR-T product	N	CAR-HT cutoff/group definition	High-risk/Low-risk n	Median follow-up	Outcomes reported
									factors/stem-cell boosts, infection, CRS, PFS/OS
**Liang<br>2025<br>United States**	Predictive model development/validation	Early ICAHT predictive model cohort	Hematologic malignancies	Commercial or investigational CAR-T	691	CAR-HEMATOTOX low vs high risk compared with eIPM Pre/eIPM Post; pre-lymphodepletion values used	Not fully extracted	Early ICAHT day 0-30; survival follow-up estimated by inverse Kaplan-Meier	Grade 3-4 early ICAHT, severe/prolonged ICAHT, model discrimination, OS
**Valtis<br>2026<br>United States**	Consortium cohort with independent investigational cohort	ROCCA adult ALL brexu-cel; MSK 1928z validation	Adult acute lymphoblastic leukemia	Brexu-cel; investigational CD19 CAR-T (1928z) in validation cohort	199	CAR HEMATOTOX low vs high risk as reported	ROCCA: HTlow 43 (22%); HThigh 156 (78%)	12-month outcomes reported	Delayed neutrophil recovery, severe infection, MRD-negative CR/CRi, OS, EFS, NRM, relapse
**Kampouri<br>2024<br>United States**	Prospective observational cohort	Fred Hutch/University of Washington CMV reactivation CARTx cohort	Hematologic malignancies; CMV-seropositive CARTx recipients	CD19/CD20- or BCMA-targeted CARTx; axi-cel, tisa-cel, liso-cel, brexu-cel, ide-cel, cilta-cel included	69	CAR-HEMATOTOX calculated as risk score/risk factor, not primary high-vs-low exposure	Not reported as high/low CAR-HT groups	Weekly CMV testing up to 12 weeks; median follow-up 81 days	CMV reactivation, CMV-specific cell-mediated immunity, CMV risk factors, infection subtype
**Stella<br>2025<br>Italy**	Prospective observational registry analysis	CART-SIE	Large B-cell lymphoma	Commercial anti-CD19 CAR-T	746 infused; CAR-HEMATOTOX evaluable 389; simpleHT evaluable 560	CAR-HEMATOTOX high risk if score ⩾2; simpleHT also evaluated	CAR-HT: low 138, high 251; simpleHT: low 465, high 95	Median follow-up 18 months; 1-year OS/PFS reported	Late grade ⩾3 ICAHT, response at day 90, OS, PFS, secondary primary malignancy, survival stratification
**Kaur<br>2026<br>United States**	Registry cohort	CIBMTR ide-cel cytopenia cohort	Multiple myeloma	Idecabtagene vicleucel	821 total; CAR-HEMATOTOX evaluable	High CAR-HEMATOTOX score⩾2 evaluated for cytopenia	Not fully extracted	Day 30 and day 100 cytopenia; survival outcomes	Day 30/day 100 cytopenias, hematologic recovery, infection/support

Author Year Country	Study design	Cohort	Disease	CAR-T product	N	CAR-HT cutoff/group definition	High-risk/Low-risk n	Median follow-up	Outcomes reported
					168	association			ive outcomes, PFS, OS
**Wiemers 2025 Germany**	Single-center observational cohort	BCMA-directed CAR-T splenomegaly cohort	Relapsed/refractory multiple myeloma	Ide-cel 35; cilta-cel 38	73	CAR-HEMATOTOX score assessed before lymphodepletion; score >1 considered high	Not fully extracted	Median follow-up: PFS 6.1 months; OS 8.2 months	Splenomegaly, thrombocytopenia, sBCMA/metabolic tumor volume, PFS, OS; CAR-HT comparator/covariate
**Zhang 2025 China**	Prospective comparative score validation	Peking University People's Hospital mixed hematologic malignancy CAR-T cohort	B-ALL, T-ALL/NHL, and multiple myeloma	B-ALL: CD19 or CD19/CD22; T-ALL/NHL: CD7; MM: BCMA; commercial and investigational products	119	CAR-HT, ALL-HT, and eIPM categories compared across diseases	CAR-HT high 80 (67.2%), low 39; ALL-HT high 56.3% of ALL/NHL patients	Follow-up collected through Aug 30, 2025; median follow-up varied by disease/risk group (about 5.9-14.2 months)	ICAHT grade/duration, prolonged neutropenia, hematologic recovery, OS, PFS, model performance
**Airosa Pardal 2025 Spain**	Retrospective immune recovery cohort	Axi-cel aggressive B-cell lymphoma cohort	Aggressive B-cell lymphoma	Axicabtagene ciloleucel	76	CAR-HEMATOTOX high vs low at lymphodepletion as reported	Low 36 (47%); high 40 (53%)	12-month PFS/OS; immune recovery during first year	Immune recovery, CD4/NK/IgG recovery, infections, PFS, OS
**Rejeski 2023 International, eight centers**	International multicenter retrospective cohort	Brexu-cel mantle cell lymphoma cohort	Relapsed/refractory mantle cell lymphoma	Brexucabtagene autoleucel	103	HTlow score 0-1 vs HThigh score ⩾2	HTlow 56; HThigh 47	1-year NRM and survival outcomes reported	Aplastic neutrophil recovery/prolonged cytopenia, severe infection, NRM, response, PFS, OS
**Rejeski 2022 International, six centers**	International multicenter retrospective cohort	CD19 CAR-T LBCL infection cohort	Relapsed/refractory large B-cell lymphoma	Axi-cel or tisa-cel	248	HTlow score 0-1 vs HThigh score ⩾2	HTlow 115 (46%); HThigh 133 (54%)	90-day infection; survival follow-up	Severe infection, bacterial infection, neutrophil recovery, hospitalization duration, PFS, OS, NRM
**Srsen**	Single-country	Adult DLBCL	Diffuse large	Commercial	14	CAR-	Not fully	Not fully	Response/remiss

Author<br>Year<br>Country	Study design	Cohort	Disease	CAR-T product	N	CAR-HT cutoff/group definition	High-risk/Low-risk n	Median follow-up	Outcomes reported
**2026<br>Slovenia**	real-world score evaluation	CAR-T Slovenia cohort	B-cell lymphoma	anti-CD19 CAR-T; tisagenlecleucel		HEMATOTOX assessed on day -5 and day 0 with EASIX-C and IBPS	extracted	extracted	ion, severe cytopenias, CRS, ICANS, predictive score performance
**Culbert<br>2025<br>United States**	Multicenter retrospective risk-factor cohort	NCI, MSKCC, and Seattle Children's B-ALL infection cohort	B-cell acute lymphoblastic leukemia	CD19_28z, CD19_BB, and CD22_BB CAR-T constructs	350	ALL-HT derivative score primary; CAR-HT also evaluated for infection risk	ALL-HT low 179, high 171; CAR-HT high 232 (88% among evaluable patients)	Severe infection through day +60	Severe infection, mild infection, infection microbiology, prior infection, ICAHT/high-risk hematotoxicity, OS
**Vic<br>2024<br>France**	National registry cohort	DESCAR-T	Large B-cell lymphoma	Commercial CD19 CAR-T	671; CAR-HEMATOTOX available for 611	CAR-HEMATOTOX score ⩾2	High 383; low 228; missing 60	Median follow-up 18.2 months	Early RBC transfusion, early platelet transfusion, late transfusion, PFS, OS, NRM
**Winkelmann<br>2025<br>International, six sites**	Multicenter observational model optimization/validation	CD19 CAR-T LBCL CAR-IMPI cohort	Large B-cell lymphoma	Axi-cel 285; liso-cel 70; tisa-cel 149	504	CAR-IMPI primary; exploratory combination with CAR-HEMATOTOX high defined as values ⩾3 and low ⩽2	Not fully extracted	Median follow-up 32.1 months overall	PFS, OS, CRS, ICANS, ICU admission, CAR-IMPI model performance; exploratory CAR-HT combination
**Barone<br>2026<br>Italy**	Prospective registry analysis	CART-SIE NRM cohort	B-cell lymphomas receiving commercial CAR-T	Axi-cel 483; tisa-cel 323; brexu-cel 126	1132 enrolled; 932 outcome-evaluable	High CAR-HEMATOTOX as pre-infusion risk factor/covariate per Rejeski definition	High 556 (67%) among patients with CAR-HT data; low n not fully extracted	Median follow-up 17.8 months (IQR 6.3-25.4)	NRM, causes of NRM, infection-related mortality, CRS, ICANS, ICAHT, survival context
**Nakamura<br>2024<br>Japan**	Single-center retrospective prediction-score cohort	Kyoto KyoTox A-score cohort	B-cell lymphoma after CAR-T	Tisa-cel 67; liso-cel 18; axi-cel 5	90 analyzed at 90 days after infusion	CAR-HEMATOTOX high vs low compared with KyoTox	CAR-HEMATOTOX high 69 (76.7%); low 21	Assessed through 90 days after infusion	Prolonged neutropenia, anemia, thrombocytopenia, prolonged

Author Year Country	Study design	Cohort	Disease	CAR-T product	N	CAR-HT cutoff/group definition	High-risk/Low-risk n	Median follow-up	Outcomes reported
						A-score	(23.3%)		cytopenia, KyoTox model performance
**Rejeski 2023 Germany/Spain**	Retrospective observational discovery study with multicenter validation cohort	CD19 CAR-T B-NHL HT10 cohort	Relapsed/refractory B-cell non-Hodgkin lymphoma	Discovery: axi-cel 23, tisa-cel 30, brexu-cel 9; validation CD19 CAR-T	Discovery n=62; validation n=125	HT10 combines baseline CAR-HEMATOTOX with day-of-fever procalcitonin; CAR-HT used as component	Not fully extracted	Early fever/infection day 0-30; validation at first fever	Early infection vs CRS, severe infection, bacterial/nonviral infection, HT10 AUC/model performance, proteomic signatures
**Xu 2026 China**	Single-center retrospective nomogram construction/validation	RRMM CAR-T nutritional status/CONUT nomogram cohort	Relapsed/refractory multiple myeloma	CAR-T regimens for RRMM; BCMA/GPRC5D-related details not fully extracted	302	CAR-HEMATOTOX used as comparator model; not primary high/low clinical-effect exposure	Not fully extracted	Not fully extracted	Prolonged grade 3/4 neutropenia >28 days, prolonged hematological toxicity composite, CONUT nomogram performance, CAR-HT AUC comparator

Note 1. Study characteristics, sample sizes, follow-up periods, CAR-HEMATOTOX definitions, and outcomes are presented as reported in the original publications. Sample sizes may differ across analyses because some scores or outcomes were available only in evaluable subsets.

Note 2. Unless otherwise specified, the original CAR-HEMATOTOX classification defines a score of 0–1 as low risk and a score of ⩾2 as high risk. Recalibrated cutoffs, derivative scores, and comparator models are reported according to the definitions used in the respective studies.

Note 3. The outcomes listed represent those evaluated or reported in each study and do not necessarily indicate inclusion in the quantitative synthesis. Outcome-level eligibility and pooling decisions are detailed in Table C3.

Note 4. "Not reported" indicates that the relevant information was not provided in the source publication. "Not fully extracted" indicates that the available report did not permit reliable extraction of the complete numerical information required for this table; no values were inferred.

Abbreviations: ALL-HT, acute lymphoblastic leukemia HEMATOTOX score; AUC, area under the curve; B-ALL, B-cell acute lymphoblastic leukemia; BCMA, B-cell maturation antigen; CAR-HEMATOTOX or CAR-HT, chimeric antigen receptor HEMATOTOX score; CAR-IMPI, CAR T-cell International Metabolic Prognostic Index; CAR-T, chimeric antigen receptor T-cell therapy; CARTx, CAR T-cell therapy; CIBMTR, Center for International Blood and Marrow Transplant Research; CMV, cytomegalovirus; CONUT, Controlling Nutritional Status; CR, complete response; CRi, complete response with incomplete count recovery; CRS, cytokine release syndrome; DLBCL, diffuse large B-cell lymphoma; EASIX-C, Endothelial Activation and Stress Index-C; EFS, event-free survival; eIPM, early ICAHT prediction model; GPRC5D, G protein-coupled receptor class C group 5 member D; HThigh, high CAR-HEMATOTOX

risk; HTlow, low CAR-HEMATOTOX risk; IBPS, Inflammation-Based Prognostic Score; ICAHT, immune effector cell-associated hematotoxicity; ICANS, immune effector cell-associated neurotoxicity syndrome; ICU, intensive care unit; IgG, immunoglobulin G; LBCL, large B-cell lymphoma; MCL, mantle cell lymphoma; MM, multiple myeloma; MRD, measurable residual disease; N, number of patients; NHL, non-Hodgkin lymphoma; NK, natural killer; NR, not reported; NRM, nonrelapse mortality; OS, overall survival; PFS, progression-free survival; R/R, relapsed or refractory; RBC, red blood cell; RRMM, relapsed or refractory multiple myeloma; sBCMA, soluble B-cell maturation antigen; T-ALL, T-cell acute lymphoblastic leukemia.

Table C2. Reporting and analytical handling of allogeneic hematopoietic stem cell transplantation in the included studies.

Author/Year	Disease setting	Allo-HSCT before CAR-T, n/N (%)	Allo-HSCT after CAR-T, n/N (%)	Outcomes evaluated	Handling in the original study	Handling in the present review
**Rejeski 2023**	RRMM treated with BCMA-directed CAR-T	4/113 (3.5%)	NR	Hematotoxicity, infection, PFS, and OS	Prior allo-SCT was reported as a baseline characteristic and compared between CAR-HEMATOTOX groups. No exclusion, transplant-specific censoring, or adjustment for prior allo-SCT was reported.	Eligible published estimates were retained without additional transplant-related adjustment.
**de Boer 2025**	R/R LBCL treated with CD19-directed CAR-T	3/245 (1.2%); all three had received both autologous and allogeneic SCT	NR	ICAHT, infection, PFS, OS, and NRM	Transplant history was reported descriptively. OS was censored at the last known follow-up; no exclusion, censoring at allo-HSCT, or allo-HSCT-specific covariate adjustment was reported.	Eligible published estimates were retained without additional transplant-related adjustment.
**Nair 2025**	B-ALL treated with investigational or commercial CAR-T	NCI cohort: 88/156 (56%); MSK cohort: 33/106 (31%); SCH cohort: 48/84 (57%)	Reported, but n/N was not provided; some patients proceeded rapidly to consolidative HSCT before day 60	Early ICAHT, neutrophil recovery, OS, and EFS	Prior HSCT was examined and was not associated with ICAHT grade. Hematotoxicity follow-up was limited to day 30 partly because of subsequent HSCT. OS and EFS were censored at the last follow-up rather than at HSCT.	The study was retained as supporting evidence; no additional transplant-related adjustment was possible.
**Valtis 2026**	Adult B-ALL treated with CD19-directed CAR-T	72/199 (36%) had previous BMT; graft type was not reported separately	32/127 (25%) responders received HCT: 10/33 CAR-HT-low and 22/94 CAR-HT-high; graft type was not reported for the full group. At least one subsequent transplant was confirmed as allogeneic.	ICAHT, infection, CRS, ICANS, OS, EFS, relapse, and NRM	Previous BMT was included in the multivariable OS model. Post-CAR-T consolidation or maintenance therapy was modeled as a time-dependent covariate for EFS. OS and EFS were otherwise censored at the last follow-up.	Outcome-specific study estimates were assessed using the prespecified eligibility criteria; no additional patient-level adjustment was performed.
**Kampouri 2024**	Hematologic malignancies treated with CD19/CD20- or BCMA-directed CAR-T	6/72 (8.3%)	NR	CMV reactivation and CMV-specific cell-mediated immunity	Prior allo-HCT was reported as a baseline characteristic. No exclusion, transplant-specific censoring, or allo-HCT-specific adjustment was reported in the final multivariable models.	The study was retained as supplementary infection-related evidence and was not used to derive an additional allo-HSCT-adjusted estimate.
**Zhang 2025**	B-ALL, T-	44/119 (37.0%)	NR	ICAHT,	Prior allo-HSCT was reported as a	The study was retained as

Author/Year	Disease setting	Allo-HSCT before CAR-T, n/N (%)	Allo-HSCT after CAR-T, n/N (%)	Outcomes evaluated	Handling in the original study	Handling in the present review
	ALL/NHL, and MM treated with CAR-T			hematologic recovery, OS, and DFS	baseline characteristic. Survival was analyzed using Kaplan-Meier curves and log-rank tests; no exclusion, transplant-specific censoring, or allo-HSCT adjustment was reported.	comparative score-validation evidence; no additional transplant-related adjustment was performed.
**Rejeski 2023 (MCL cohort)**	R/R MCL treated with brexucabtagene autoleucel	Reported; n/N not available in the main article	NR	Hematotoxicity, infection, PFS, OS, and NRM	Prior allogeneic or autologous SCT was compared between CAR-HEMATOTOX groups and did not differ significantly. No allo-SCT-specific exclusion, censoring, or adjustment was reported.	Eligible published estimates were retained without additional transplant-related adjustment.
**Sršen 2026**	Adult DLBCL or PMBCL treated with tisagenlecleucel	0/14 (0%) reported	2/14 (14.3%)	Response, relapse, survival, CRS, ICANS, and cytopenias	Post-CAR-T allo-SCT was listed among subsequent treatments for two individual patients. The cohort analyses were descriptive; no exclusion, transplant-specific censoring, or adjustment was reported.	The study was retained as descriptive score-evaluation evidence and was not used to derive an allo-SCT-adjusted estimate.
**Culbert 2025**	Pediatric and adult B-ALL treated with CAR-T	171/350 (49%)	NR	Severe infection through day 60 after CAR-T	Prior HSCT was reported and compared across risk groups. It was not retained in the reported multivariable severe-infection model. No exclusion or transplant-specific censoring was described.	The study was retained as supplementary infection-related evidence; no additional transplant-related adjustment was performed.
**Vic 2024**	R/R LBCL treated with commercial CD19-directed CAR-T	8/671 (1.2%)	NR	Early and late transfusion requirements, PFS, and OS	Prior allo-HSCT was reported as a baseline characteristic. Transfusion follow-up was censored at relapse, initiation of a new treatment, or death, not specifically at allo-HSCT. No allo-HSCT adjustment was reported.	Eligible transfusion estimates were retained without additional transplant-related adjustment.

Note 1. Data are shown only for studies that explicitly reported allo-HSCT or reported HCT/BMT information directly relevant to the handling of allo-HSCT. The remaining included studies did not report allo-HSCT separately.

Note 2. “Before CAR-T” refers to transplantation before the index CAR-T infusion. “After CAR-T” refers to consolidative or subsequent transplantation during post-infusion follow-up.

Note 3. When an article reported HCT or BMT without identifying the graft as allogeneic, the transplant type was recorded as not reported and was not inferred.

Note 4. The present review used published aggregate estimates and did not have access to individual patient data; therefore, no additional patient-level exclusion, censoring, or covariate adjustment for allo-HSCT was undertaken.

Abbreviations: allo-HSCT, allogeneic hematopoietic stem cell transplantation; B-ALL, B-cell acute lymphoblastic leukemia; BMT, bone marrow transplantation; CAR-T, chimeric antigen receptor T-cell therapy; CMV, cytomegalovirus; CRS, cytokine release syndrome; DFS, disease-free survival; EFS, event-free survival; HCT, hematopoietic cell transplantation; ICAHT, immune effector cell-associated hematotoxicity; ICANS, immune effector cell-associated neurotoxicity syndrome; LBCL, large B-cell lymphoma; MCL, mantle cell lymphoma; MM, multiple myeloma; NR, not reported; NRM, nonrelapse mortality; OS, overall survival; PFS, progression-free survival; PMBCL, primary mediastinal B-cell lymphoma; R/R, relapsed or refractory; RRMM, relapsed or refractory multiple myeloma; SCT, stem cell transplantation.

Table C3. Outcome-Level Extraction and Pooling Decision.

Author/Year	CAR-HEMATOTOX cutoff	High-risk Group definition	Reference group	Outcome definition	Assessment time point	Reported effect measure	Use in quantitative synthesis
**Rejeski 2021**	Original CAR-HEMATOTOX score; high risk generally score⩾2 vs low 0-1/<2.	CAR-HT high-risk/model-positive group, generally score≥2.	low-risk group	prolonged cytopenia: duration of severe neutropenia	study-specific; see source evidence	median days; reported value 12 vs 5.5	Not included in quantitative synthesis; retained as model-development and contextual evidence because the endpoint and training/validation structure differed from the pooled clinical-effect estimates.
**Rejeski 2021**	Original CAR-HEMATOTOX score; high risk generally score ⩾ 2 vs low 0-1/<2.	CAR-HT high-risk/model-positive group, generally score≥2.	low-risk group	prolonged cytopenia: severe neutropenia ⩾14 days	study-specific; see source evidence	OR	Not included in quantitative synthesis; retained as model-development evidence because this estimate was not the most comparable hematotoxicity endpoint for cross-study pooling.
**Rejeski 2023**	HTlow score 0-1 vs HThigh score ⩾2.	HThigh score≥2.	HTlow score 0-1 / low-risk group.	CRS: severe CRS	study-specific severe CRS reporting window	raw event-count OR	Included in supplementary quantitative synthesis of severe CRS; interpreted as hypothesis-generating because evidence was sparse and the pooled association was not statistically significant.
**Rejeski 2023**	HTlow score 0-1 vs HThigh score ⩾2.	HThigh score≥2.	low-risk group	ICANS: grade≥3 ICANS	study-specific; see source evidence	reported event rates; OR	Included in supplementary quantitative synthesis of severe ICANS; interpreted as hypothesis-generating because evidence was sparse and the pooled association was not statistically significant.
**Rejeski 2023**	HTlow score 0-1 vs HThigh score⩾ 2.	HThigh score≥2.	low-risk group	NRM/TRM: 1-year non-relapse mortality	study-specific; see source evidence	risk difference / event rate; reported value 13% vs 2%	Not pooled because fewer than three compatible estimates were available for non-relapse or treatment-related mortality.
**Rejeski 2023**	HTlow score 0-1 vs HThigh score ⩾2.	HThigh score≥2.	low-risk group	OS: overall survival	study-specific; see source evidence	aHR	Included in exploratory quantitative synthesis of overall survival using hazard-ratio estimates.
**Rejeski 2023**	HTlow score 0-1 vs HThigh score ⩾2.	HThigh score≥2.	low-risk group	PFS: progression-free survival	study-specific; see source evidence	aHR	Included in exploratory quantitative synthesis of progression-free survival using hazard-ratio estimates.
**Rejeski 2023**	HTlow score 0-1 vs HThigh score ⩾2.	HThigh score≥2.	HTlow score 0-1 / low-risk group.	hospitalization/resource: early RBC transfusion	early post-infusion supportive-care window as reported	OR; raw event-count/reconstructed estimate	Included in supplementary quantitative synthesis of early red blood cell transfusion; interpreted as supportive-care evidence rather than evidence for hospitalization duration.
**Rejeski 2023**	HTlow score 0-1	HThigh score≥2.	HTlow score 0-1 / low-risk	hospitalization/resource: early platelet	early post-infusion	OR; raw event-count/reconstructed	Included in supplementary quantitative synthesis of early platelet transfusion;

Author/Year	CAR-HEMATOTOX cutoff	High-risk Group definition	Reference group	Outcome definition	Assessment time point	Reported effect measure	Use in quantitative synthesis
	vs HThigh score⩾ 2.		group.	transfusion	supportive-care window as reported	estimate	interpreted as supportive-care evidence rather than evidence for hospitalization duration.
**Rejeski 2023**	HTlow score 0-1 vs HThigh score⩾ 2.	HThigh score≥2.	low-risk group	infection: severe infection	study-specific; see source evidence	reported event rates; reconstructed OR	Included in exploratory quantitative synthesis of severe infection after conversion from reported group-level event data.
**Rejeski 2023**	HTlow score 0-1 vs HThigh score⩾ 2.	HThigh score≥2.	low-risk group	prolonged cytopenia: aplastic neutrophil recovery phenotype	study-specific; see source evidence	aOR	Included in exploratory quantitative synthesis of hematotoxicity/ICAHT using odds-ratio estimates.
**de Boer 2025**	Original CAR-HT cutoff ⩾2 vs <2; recalibrated cutoff ⩾3 also evaluated.	Original HThigh score≥2 for the pooled original-score analyses.	low-risk group	ICAHT: early ICAHT grade≥3	study-specific; see source evidence	OR	Not pooled because another hematotoxicity endpoint from the same study provided the more comparable estimate for cross-study synthesis.
**de Boer 2025**	Original CAR-HT cutoff ⩾2 vs <2; recalibrated cutoff ⩾3 also evaluated.	Original HThigh score≥2 for the pooled original-score analyses.	low-risk group	ICAHT: late ICAHT grade≥3	study-specific; see source evidence	OR	Included in exploratory quantitative synthesis of hematotoxicity/ICAHT using odds-ratio estimates.
**de Boer 2025**	Original CAR-HT cutoff ⩾2 vs <2; recalibrated cutoff ⩾3 also evaluated.	Original HThigh score≥2 for the pooled original-score analyses.	low-risk group	OS: overall survival	study-specific; see source evidence	HR	Included in exploratory quantitative synthesis of overall survival using hazard-ratio estimates.
**de Boer 2025**	Original CAR-HT cutoff ⩾2 vs <2; recalibrated cutoff ⩾3 also evaluated.	Original HThigh score≥2 for the pooled original-score analyses.	low-risk group	PFS: progression-free survival	study-specific; see source evidence	HR	Included in exploratory quantitative synthesis of progression-free survival using hazard-ratio estimates.
**de Boer 2025**	Original CAR-HT cutoff ⩾2 vs <2; recalibrated cutoff ⩾3 also evaluated.	Original HThigh score≥2 for the pooled original-score analyses.	low-risk group	infection: severe infections	study-specific; see source evidence	OR	Included in exploratory quantitative synthesis of severe infection using odds-ratio estimates.

Author/Year	CAR-HEMATOTOX cutoff	High-risk Group definition	Reference group	Outcome definition	Assessment time point	Reported effect measure	Use in quantitative synthesis
**de Boer 2025**	Original CAR-HT cutoff⩾2 vs <2; recalibrated cutoff ⩾3 also evaluated.	Original HThigh score≥2 for the pooled original-score analyses.	low-risk group	prolonged cytopenia: clinically significant neutropenia	study-specific; see source evidence	OR	Not pooled because another hematotoxicity endpoint from the same study provided the more comparable estimate for cross-study synthesis.
**Nair 2025**	Original CAR-HT applied but classified nearly 90% as high risk; ALL-HT derivative score developed for B-ALL.	Original CAR-HT high risk and ALL-HT high-risk derivative group as reported.	Original CAR-HT low/reference group or ALL-HT low group as reported.	ICAHT, severe/prolonged neutropenia, infection, response, OS, and ALL-HT model performance.	study-specific / as reported	Model-performance and score-validation metrics; no compatible original high-vs-low clinical-effect estimate extracted.	Not pooled because the study primarily developed or validated a derivative score and did not provide a compatible original CAR-HEMATOTOX high-versus-low clinical-effect estimate.
**Cook 2026**	CAR-HT low <2 vs high ⩾2.	CAR-HT score≥2.	low-risk group	CRS: CRS	study-specific; see source evidence	OR	Not pooled because the outcome definition, timing, and possible cohort overlap limited compatibility with the quantitative synthesis.
**Cook 2026**	CAR-HT low <2 vs high⩾2.	CAR-HT score≥2.	low-risk group	infection: infection	study-specific; see source evidence	OR	Not pooled because the infection endpoint was not sufficiently compatible with the severe-infection odds-ratio synthesis.
**Liang 2025**	CAR-HEMATOTOX low vs high risk compared with eIPM Pre/eIPM Post; exact high/low cutoff not fully extracted.	CAR-HT high-risk category as reported in score-comparison analysis.	CAR-HT low/reference category as reported.	Early grade 3-4 ICAHT, severe/prolonged ICAHT, model discrimination, and OS.	study-specific / as reported	Prediction-model performance and validation metrics.	Not pooled because the study focused on prediction-model performance and did not provide a compatible original CAR-HEMATOTOX high-versus-low clinical-effect estimate.
**Valtis 2026**	CAR HEMATOTOX low vs high risk as reported in adult ALL cohort.	HT high-risk group as reported.	HT low/reference group as reported.	CRS: severe CRS	study-specific severe CRS reporting window	raw event-count OR	Included in supplementary quantitative synthesis of severe CRS; interpreted as hypothesis-generating because evidence was sparse and the pooled association was not statistically significant.
**Valtis 2026**	CAR HEMATOTOX low vs high risk as reported in adult ALL cohort.	HT high-risk group as reported.	HT low/reference group as reported.	ICANS: severe ICANS	study-specific severe ICANS reporting window	raw event-count OR	Included in supplementary quantitative synthesis of severe ICANS; interpreted as hypothesis-generating because evidence was sparse and the pooled association was not statistically significant.
**Valtis 2026**	CAR	HT high-risk	as reported	OS: overall survival	study-	HR	Not pooled because the survival estimate

Author/Year	CAR-HEMATOTOX cutoff	High-risk Group definition	Reference group	Outcome definition	Assessment time point	Reported effect measure	Use in quantitative synthesis
	HEMATOTOX low vs high risk as reported in adult ALL cohort.	group as reported.			specific; see source evidence		had incomplete interval reporting and lower extraction confidence.
**Valtis 2026**	CAR HEMATOTOX low vs high risk as reported in adult ALL cohort.	HT high-risk group as reported.	as reported	infection: severe infections	study-specific; see source evidence	reported event rates; reconstructed raw OR	Included in exploratory quantitative synthesis of severe infection using a reconstructed odds-ratio estimate from reported event proportions and denominators.
**Valtis 2026**	CAR HEMATOTOX low vs high risk as reported in adult ALL cohort.	HT high-risk group as reported.	as reported	response: MRD-negative CR/CRi	study-specific; see source evidence	OR	Not pooled because response definitions and effect directions were heterogeneous across studies.
**Kampouri 2024**	CAR-HEMATOTOX calculated as risk score/risk factor, not primary high-vs-low exposure.	No extractable CAR-HT high-risk group for compatible clinical-effect pooling.	No compatible low/reference group extracted.	CMV reactivation, CMV-specific immunity, and infection risk factors after CAR-T.	study-specific / as reported	Risk-factor/model covariate evidence; no compatible high-vs-low effect metric extracted.	Not pooled because CAR-HEMATOTOX was not reported as a compatible high-versus-low clinical-effect exposure.
**Stella 2025**	CAR-HEMATOTOX high risk if score ⩾2; simpleHT also evaluated.	CAR-HT score≥2.	as reported	ICAHT: late ICAHT grade≥3	study-specific; see source evidence	OR	Included in exploratory quantitative synthesis of hematotoxicity/ICAHT using odds-ratio estimates.
**Stella 2025**	CAR-HEMATOTOX high risk if score≥2; simpleHT also evaluated.	CAR-HT score≥2.	as reported	OS: 1-year overall survival	study-specific; see source evidence	event/rate comparison; reported value 62% vs 78%	Not pooled because the outcome format and timepoint were not compatible with the survival hazard-ratio synthesis.
**Stella 2025**	CAR-HEMATOTOX high risk if score≥2; simpleHT also evaluated.	CAR-HT score≥2.	as reported	response: complete response rate at day 90	study-specific; see source evidence	OR	Not pooled because response definitions and effect directions were heterogeneous across studies.
**Stella 2025**	CAR-HEMATOTOX high risk if score≥2; simpleHT also	CAR-HT score≥2.	as reported	response: overall response rate at day 90	study-specific; see source evidence	OR	Not pooled because response definitions and effect directions were heterogeneous across studies.

Author/Year	CAR-HEMATOTOX cutoff	High-risk Group definition	Reference group	Outcome definition	Assessment time point	Reported effect measure	Use in quantitative synthesis
	evaluated.						
**Kaur 2026**	High CAR-HEMATOTOX score≥2 evaluated for cytopenia association.	CAR-HT score≥2 / high-risk subgroup where available.	as reported	prolonged cytopenia: day 30 cytopenia association with CAR-HEMATOTOX	study-specific; see source evidence	not extractable; reported value not reliably extracted	Not pooled because the reported cytopenia association was not available as a compatible high-versus-low effect estimate.
**Wiemers 2025**	CAR-HEMATOTOX score assessed before lymphodepletion; score >1 considered high.	CAR-HT score >1.	CAR-HT score ≤1 / as reported.	Splenomegaly, thrombocytopenia, sBCMA/metabolic tumor volume, PFS, OS, and CAR-HT covariate/comparator evidence.	study-specific / as reported	Covariate/model-comparison evidence; no compatible clinical-effect estimate extracted.	Not pooled because CAR-HEMATOTOX was used mainly as a covariate or comparator rather than as a compatible high-versus-low clinical-effect exposure.
**Zhang 2025**	CAR-HT, ALL-HT, and eIPM categories compared across diseases.	CAR-HT high category as reported; ALL-HT/eIPM high categories also evaluated.	Low/reference categories as reported.	ICAHT grade/duration, prolonged neutropenia, hematologic recovery, OS, PFS, and model performance.	study-specific / as reported	Comparative validation and model-performance metrics.	Not pooled because the study was a comparative score-validation analysis with mixed or derivative score evidence.
**Airosa Pardal 2025**	CAR-HEMATOTOX high vs low at lymphodepletion as reported.	High CAR-HEMATOTOX group as reported.	low-risk group	OS: overall survival	study-specific; see source evidence	HR	Included in exploratory quantitative synthesis of overall survival using hazard-ratio estimates.
**Airosa Pardal 2025**	CAR-HEMATOTOX high vs low at lymphodepletion as reported.	High CAR-HEMATOTOX group as reported.	low-risk group	PFS: 12-month progression-free survival	study-specific; see source evidence	HR	Included in exploratory quantitative synthesis of progression-free survival using hazard-ratio estimates.
**Rejeski 2023**	HTlow score 0-1 vs HThigh score≥2.	HThigh score≥2.	HTlow score 0-1 / low-risk group.	CRS: severe CRS	study-specific severe CRS reporting window	raw event-count OR	Included in supplementary quantitative synthesis of severe CRS; interpreted as hypothesis-generating because evidence was sparse and the pooled association was not statistically significant.
**Rejeski 2023**	HTlow score 0-1 vs HThigh score≥2.	HThigh score≥2.	HTlow score 0-1 / low-risk group.	ICANS: severe ICANS	study-specific severe ICANS reporting window	raw event-count OR	Included in supplementary quantitative synthesis of severe ICANS; interpreted as hypothesis-generating because evidence was sparse and the pooled association was not statistically significant.
**Rejeski 2023**	HTlow score 0-1 vs HThigh	HThigh score≥2.	low-risk group	NRM/TRM: 1-year non-relapse mortality	study-specific;	event/rate comparison; reported value 17% vs	Not pooled because fewer than three compatible estimates were available for

Author/Year	CAR-HEMATOTOX cutoff	High-risk Group definition	Reference group	Outcome definition	Assessment time point	Reported effect measure	Use in quantitative synthesis
	score≥2.				see source evidence	4.6%	non-relapse or treatment-related mortality.
**Rejeski 2023**	HTlow score 0-1 vs HThigh score≥2.	HThigh score≥2.	low-risk group	OS: overall survival	study-specific; see source evidence	aHR	Included in exploratory quantitative synthesis of overall survival using hazard-ratio estimates.
**Rejeski 2023**	HTlow score 0-1 vs HThigh score≥2.	HThigh score≥2.	low-risk group	PFS: progression-free survival	study-specific; see source evidence	aHR	Included in exploratory quantitative synthesis of progression-free survival using hazard-ratio estimates.
**Rejeski 2023**	HTlow score 0-1 vs HThigh score≥2.	HThigh score≥2.	HTlow score 0-1 / low-risk group.	hospitalization/resource: early RBC transfusion	early post-infusion supportive-care window as reported	OR; raw event-count/reconstructed estimate	Included in supplementary quantitative synthesis of early red blood cell transfusion; interpreted as supportive-care evidence rather than evidence for hospitalization duration.
**Rejeski 2023**	HTlow score 0-1 vs HThigh score≥2.	HThigh score≥2.	HTlow score 0-1 / low-risk group.	hospitalization/resource: early platelet transfusion	early post-infusion supportive-care window as reported	OR; raw event-count/reconstructed estimate	Included in supplementary quantitative synthesis of early platelet transfusion; interpreted as supportive-care evidence rather than evidence for hospitalization duration.
**Rejeski 2023**	HTlow score 0-1 vs HThigh score≥2.	HThigh score≥2.	low-risk group	infection: severe infections	study-specific; see source evidence	aOR	Included in exploratory quantitative synthesis of severe infection using odds-ratio estimates.
**Rejeski 2023**	HTlow score 0-1 vs HThigh score≥2.	HThigh score≥2.	low-risk group	prolonged cytopenia: aplastic neutrophil recovery phenotype	study-specific; see source evidence	aOR	Included in exploratory quantitative synthesis of hematotoxicity/ICAHT using odds-ratio estimates.
**Rejeski 2022**	HTlow score 0-1 vs HThigh score≥2.	HThigh score≥2.	as reported	OS: overall survival	study-specific; see source evidence	median comparison; reported value 9.1 months vs not reached	Not pooled because the survival result was reported as a median comparison rather than a compatible hazard-ratio estimate.
**Rejeski 2022**	HTlow score 0-1 vs HThigh score≥2.	HThigh score≥2.	as reported	PFS: progression-free survival	study-specific; see source evidence	median comparison; reported value 3.4 vs 12.6 months	Not pooled because the progression-free survival result was reported as a median comparison rather than a compatible hazard-ratio estimate.
**Rejeski 2022**	HTlow score 0-1 vs HThigh score≥2.	HThigh score≥2.	as reported	infection: bacterial infections	study-specific; see source evidence	aHR	Not pooled because bacterial infection was not sufficiently aligned with the severe-infection endpoint used for quantitative synthesis.

Author/Year	CAR-HEMATOTOX cutoff	High-risk Group definition	Reference group	Outcome definition	Assessment time point	Reported effect measure	Use in quantitative synthesis
**Rejeski 2022**	HTlow score 0-1 vs HThigh score≥2.	HThigh score≥2.	HTlow score 0-1 / as reported.	infection: grade≥3 severe infection	90-day infection window / as reported	aOR; supplemental extraction recorded 7.70 (95% CI 3.40 to 17.30)	Included in exploratory quantitative synthesis of severe infection using an odds-ratio estimate.
**Rejeski 2022**	HTlow score 0-1 vs HThigh score≥2.	HThigh score≥2.	as reported	infection: severe infections	study-specific; see source evidence	aHR	Not pooled because a more compatible odds-ratio estimate from the same study was used for the severe-infection synthesis.
**Srsen 2026**	CAR-HEMATOTOX assessed on day -5 and day 0 with EASIX-C and IBPS.	High-risk score groups not fully extracted for compatible pooling.	Reference groups not fully extracted.	Response/remission, severe cytopenias, CRS, ICANS, and predictive-score performance.	study-specific / as reported	Small score-evaluation/descriptive metrics.	Not pooled because the cohort was small and the study focused primarily on score evaluation rather than compatible high-versus-low clinical-effect estimation.
**Culbert 2025**	ALL-HT derivative score primary; CAR-HT also evaluated for infection risk.	ALL-HT high-risk group primary; CAR-HT high group reported for subset.	ALL-HT or CAR-HT low/reference group as reported.	Severe infection through day +60, mild infection, microbiology, prior infection, ICAHT/high-risk hematotoxicity, and OS.	study-specific / as reported	Risk-factor/model evidence; no compatible original CAR-HT pooled estimate extracted.	Not pooled because the primary exposure was a derivative ALL-HEMATOTOX score and compatibility with original CAR-HEMATOTOX synthesis was limited.
**Vic 2024**	CAR-HEMATOTOX score≥2.	CAR-HT score≥2.	low-risk group	hospitalization/resource: early RBC transfusion	study-specific; see source evidence	aOR	Included in supplementary quantitative synthesis of early red blood cell transfusion; interpreted as supportive-care evidence rather than evidence for hospitalization duration.
**Vic 2024**	CAR-HEMATOTOX score≥2.	CAR-HT score≥2.	low-risk group	hospitalization/resource: early platelet transfusion	study-specific; see source evidence	aOR	Included in supplementary quantitative synthesis of early platelet transfusion; interpreted as supportive-care evidence rather than evidence for hospitalization duration.
**Vic 2024**	CAR-HEMATOTOX score≥2.	CAR-HT score≥2.	low-risk group	hospitalization/resource: late transfusion	study-specific; see source evidence	aOR	Not pooled because late transfusion was not part of the prespecified early transfusion supportive-care synthesis.
**Winkelmann 2025**	CAR-IMPI primary; exploratory combination with CAR-HT high defined as≥3 and low ≤2.	CAR-HT high≥3 in exploratory combination.	CAR-HT low ≤2 in exploratory combination.	PFS, OS, CRS, ICANS, ICU admission, CAR-IMPI model performance, and exploratory CAR-HT combination.	study-specific / as reported	Model-performance/prognostic-index metrics.	Not pooled because the study focused on a metabolic prognostic index and did not provide a compatible original CAR-HEMATOTOX high-versus-low clinical-effect estimate.
**Barone 2026**	High CAR-	High CAR-HT	Low/reference	NRM, causes of NRM,	study-	Registry risk-	Not pooled because of cohort overlap

Author/Year	CAR-HEMATOTOX cutoff	High-risk Group definition	Reference group	Outcome definition	Assessment time point	Reported effect measure	Use in quantitative synthesis
	HEMATOTOX as pre-infusion risk factor/covariate per Rejeski definition.	group as reported among patients with CAR-HT data.	group not fully extracted.	infection-related mortality, CRS, ICANS, ICAHT, and survival context.	specific / as reported	factor/covariate evidence.	concerns and insufficient compatible estimates for non-relapse or treatment-related mortality.
**Nakamura 2024**	CAR-HEMATOTOX high vs low compared with KyoTox A-score.	CAR-HT high group as reported.	CAR-HT low group as reported.	Prolonged neutropenia, anemia, thrombocytopenia, prolonged cytopenia, and KyoTox model performance.	study-specific / as reported	Model-performance/comparator metrics.	Not pooled because the study focused on a comparator prediction model rather than a compatible original CAR-HEMATOTOX clinical-effect estimate.
**Rejeski 2023**	HT10 combines baseline CAR-HEMATOTOX with day-of-fever procalcitonin; CAR-HT used as component.	HT10/model-positive group, not a pure baseline CAR-HT high group.	as reported	infection: early infections during CRS	study-specific; see source evidence	AUC; reported value 0.87	Retained as supplementary model-performance evidence; not eligible for clinical-effect meta-analysis.
**Xu 2026**	CAR-HEMATOTOX used as comparator model; not primary high/low clinical-effect exposure.	Not applicable for primary clinical-effect pooling.	as reported	prolonged cytopenia: prolonged hematological toxicity	study-specific; see source evidence	AUC; reported value 0.706 (95% CI 0.702 to 0.713)	Retained as supplementary model-performance evidence; not eligible for clinical-effect meta-analysis.
**Reynolds 2025**	Bedside CAR-HEMATOTOX high vs low; exact cutoff not fully stored in the original 23-study extraction table.	High CAR-HT group as reported.	Low CAR-HT/reference group as reported.	infection: grade≥3 severe infection	day 0-90 infection; early and late infection windows also reported	OR; supplemental extraction recorded 8.67 (95% CI 2.30 to 32.68)	Included in exploratory quantitative synthesis of severe infection as an independent additional estimate.

Note 1. Each row represents an outcome-level estimate or a distinct outcome assessment. Consequently, a single study may contribute multiple rows.

Note 2. Quantitative synthesis was performed only when an outcome–effect-measure combination included at least three sufficiently comparable independent estimates. Comparability was assessed with respect to the study population, clinical setting, CAR-HEMATOTOX definition, outcome definition, assessment window, and effect measure.

Note 3. When multiple estimates from the same study or cohort were available for the same outcome, the estimate most consistent with the prespecified outcome definition and the other contributing studies was selected to avoid double counting. Other estimates were retained as contextual or narrative evidence.

Note 4. Adjusted estimates were preferred when they were reported and sufficiently compatible. Reconstructed odds ratios were calculated from published group-level event counts or proportions when the necessary data were available. Odds ratios and hazard ratios were analyzed separately, and fixed-time or event-based outcomes were not combined with time-to-event outcomes.

Note 5. "Study-specific; see source evidence" indicates that the assessment window or outcome definition was retained as reported in the original publication and was not further standardized when the available information did not support reliable harmonization.

Note 6. Estimates excluded from quantitative synthesis were retained in the narrative synthesis or as model-development, validation, comparator, or contextual evidence. The principal reason for each pooling decision is provided in the final column.

Abbreviations: aHR, adjusted hazard ratio; aOR, adjusted odds ratio; ALL-HT, acute lymphoblastic leukemia HEMATOTOX score; AUC, area under the curve; CAR-HEMATOTOX or CAR-HT, chimeric antigen receptor HEMATOTOX score; CI, confidence interval; CMV, cytomegalovirus; CR, complete response; CRi, complete response with incomplete count recovery; CRS, cytokine release syndrome; EASIX-C, Endothelial Activation and Stress Index-C; EFS, event-free survival; eIPM, early ICAHT prediction model; HR, hazard ratio; HT10, CAR-HEMATOTOX-based infection prediction model incorporating procalcitonin at first fever; HThigh, high CAR-HEMATOTOX risk; HTlow, low CAR-HEMATOTOX risk; IBPS, Inflammation-Based Prognostic Score; ICAHT, immune effector cell-associated hematotoxicity; ICANS, immune effector cell-associated neurotoxicity syndrome; ICU, intensive care unit; MRD, measurable residual disease; NRM, nonrelapse mortality; OR, odds ratio; OS, overall survival; PFS, progression-free survival; RBC, red blood cell; sBCMA, soluble B-cell maturation antigen; TRM, treatment-related mortality.
